# Birth-cohort estimates of smoking initiation and prevalence in 20^th^ century Australia: Synthesis of data from 33 surveys and 385,810 participants

**DOI:** 10.1371/journal.pone.0250824

**Published:** 2021-05-21

**Authors:** Pavla Vaneckova, Stephen Wade, Marianne Weber, John M. Murray, Paul Grogan, Michael Caruana, Emily Banks, Karen Canfell

**Affiliations:** 1 The Daffodil Centre, The University of Sydney, a joint venture with Cancer Council NSW, Sydney, NSW, Australia; 2 School of Mathematics and Statistics, UNSW, Sydney, NSW, Australia; 3 National Centre for Epidemiology and Population Health, Research School of Population Health, Australian National University, Canberra, ACT, Australia; 4 The Sax Institute, Sydney, NSW, Australia; 5 Prince of Wales Clinical School, University of New South Wales, Sydney, NSW, Australia; University of Calfornia San Francisco, UNITED STATES

## Abstract

The aim of our study was to quantify sex-specific patterns of smoking prevalence and initiation in 10-year birth cohorts from 1910 to 1989 in Australia. We combined individual data of 385,810 participants from 33 cross-sectional surveys conducted between 1962 and 2018. We found that age-specific smoking prevalence varied considerably between men and women within birth cohorts born before 1960. The largest difference was observed in the earliest cohort (1910–1919), with up to 37.7% point greater proportion of current smokers in men than in women. In subsequent cohorts, the proportion decreased among men, but increased among women, until there was no more than 7.4% point difference in the 1960–69 birth cohort. In the 1970–79 and 1980–89 cohorts, smoking among men marginally increased, but the proportion was at most ~11.0% points higher than women. Our analysis of initiation indicated that many women born before the 1930s who smoked commenced smoking after age 25 years (e.g., ~27% born in 1910–19); compared to at most 8% of men in any birth cohort. The earliest birth cohort (1910–1919) had the greatest difference in age at initiation between sexes; 26.6 years in women versus 19.0 in men. In later cohorts, male and female smokers initiated increasingly earlier, converging in the 1960–69 cohort (17.6 and 17.8 years, respectively). While 22.9% of men and 8.4% of women initiated smoking aged < = 15 in the 1910–1919 cohort, in the latest cohort (1980–89) the reverse was true (21.4% and 28.8% for men and women, respectively). Marked differences in smoking prevalence and age at initiation existed between birth cohorts of Australian men and women born before 1960; after this, sex-specific trends in prevalence and initiation were similar. Understanding these patterns may inform the evaluation of tobacco control policies and the targeting of potential interventions for exposed populations such as lung cancer screening.

## Introduction

Tobacco use imposes a large burden of disease and premature death and remains a major risk factor for many non-communicable diseases, such as cancer, diabetes, and cardiovascular and chronic lung diseases. In Australia, tobacco use was estimated to cause 1.7 million hospital inpatient episodes, incurring $19.2 billion in tangible costs, and to kill approximately 21,000 Australians (equivalent to 13.3% of all deaths) in the 2015–2016 financial year [1, 2]. In 2015, tobacco use was also responsible for 9.3% of the total national burden of disease, the single risk factor responsible for the greatest disease burden and the leading risk factor for both men and women aged 65–84 years [2].

Tobacco use remains Australia’s primary preventable cause of ill health and death, with 11.0% (2.3 million) of those aged 14 years and over still smoking on a daily basis [3, 4]. Over the past 5–6 decades, Australia has worked to reduce tobacco use through multifaceted approaches to tobacco control, including taxation, bans on advertising, smoke-free legislation, packaging restrictions, point-of-sale legislation, and anti-tobacco mass media campaigns. In 1983 Australia was the first country to use state-wide anti-smoking campaigns and in 2012 became the first to introduce plain packaging [5–7]. Ongoing reforms, including Australia’s first major National Tobacco Campaign in the late 1990s, significant excise increases and plain packaging (associated with ~25% of the total prevalence decline over the nearly three years of the post-implementation period [8]), coincided with a reduction in prevalence of current daily smoking among adults between 1995 and 2017/18 from ~24% to ~14%, now one of the lowest levels in the world [3, 4, 9–11]. However, the recently-conducted national cross-sectional survey reported no significant change in daily smoking rates among some Australian subgroups [4, 12].

Furthermore, as smoking prevalence approaches <10%, the ability to detect significant declines becomes more difficult and smoking levels in targeted population sub-groups with higher than average rates of smoking may be of interest. For example, Australians aged 40–59 years continue to have relatively high prevalences of daily smoking (~16%) regardless of anti-smoking interventions [4, 10, 11, 13, 14]. Differences in smoking prevalence by age, birth cohort, period and sex may reflect generational differences in exposure to tobacco promotion and control interventions, and smoking-related social norms at different periods over the life-course [15–17]. Hence, analyses of smoking trends within the context of birth cohorts could further advance our understanding of the patterns of smoking exposure and tobacco control [18].

Historically, data on smoking trends in Australia have been derived from nationally representative, periodically-conducted, population-based cross-sectional surveys, such as the National Drug Strategy Household Survey (NDSHS) administered by the Australian Institute of Health and Welfare [19], Australian/National Health Surveys (NHS) conducted by Australian Bureau of Statistics (ABS) [20] and earlier, the Risk Factor Prevalence Surveys (RFPS) by the National Heart Foundation as well as surveys overseen by the Cancer Council Victoria (CCV). However, results of discrete cross-sectional survey studies, such as these, are limited to a single point in time and typically only report the smoking behaviour of a group of people at a specific age. For that reason, they do not provide a complete picture of changes in smoking behaviours for different birth cohorts as they age. One way of estimating trends in smoking behaviours for birth cohorts is to utilise individual data collected in multiple cross-sectional surveys to construct life-course smoking histories for participants up to the date they were interviewed and then assign them to specific birth cohorts. This method has been used previously in the US and the UK, and on a smaller scale in Australia [21–23]. The US National Cancer Institute Cancer Intervention and Surveillance Modelling Network (CISNET) used individual smoking histories from multiple cross-sectional National Health Interview Surveys to construct birth cohorts for those born from the early 1900s to the 1980s [21, 24]. These birth cohorts were then used to analyse differential smoking patterns among African American and White birth cohorts in the US [24], and the effect of education and socio-economic status on sex- and cohort-specific smoking behaviour [25]. Other birth cohort studies of smoking patterns have been used to investigate whether changes in cigarette design were associated with changes in specific types of lung cancer [26], and how the changes over time in socio-economic correlates of smoking affect early menopause among women [27]. Histories of life-course smoking built from cross-sectional surveys have also been used as inputs into large microsimulation models to predict future tobacco and lung cancer mortality patterns under different tobacco control scenarios in the US [25, 28–31].

Australian studies presenting birth cohort-specific smoking behaviours are few, and span relatively short periods of time, thus offering a limited understanding of the trends in smoking rates of different birth cohorts. Given Australia’s unique tobacco control context, more detailed analyses are likely to be informative both locally and internationally. Hence, we analysed data on smoking prevalence and initiation for 10-year Australian birth cohorts born from 1910 onwards. These are the first birth-cohort specific estimates of smoking exposure in Australia spanning most of the 20^th^ century combining multiple nationally representative surveys. The proposed research may help current tobacco control efforts by identifying high-risk population subgroups for anti-tobacco message targeting or cessation interventions. The results of our study will be critical to the development of simulation models of tobacco-related disease and mortality in Australia.

## Data and methods

We acquired individual-level data from the ABS, Australian Data Archive (ADA) and the Cancer Council Victoria for five series of cross-sectional surveys: the Australian/National Health Survey (NHS), the National Drug Strategy Household Survey (NDSHS), the Risk Factor Prevalence Study (RFPS), the Australian Gallup Polls (AGP) and the Cancer Council Victoria (CCV) surveys, a total of 33 exclusive surveys conducted between 1962 and 2018 [19, 20]. These surveys collected information about the sex, age and smoking behaviour of participants. The NHS, NDSHS and CCV are nationally representative surveys, the AGP consist of quota-sampled telephone participants from the Australian population, the RFPS were conducted in capital cities only, and the Social Issues in Australia 1985 and National Campaign Against Drug Abuse Social Issues in Australia Survey 1991 were conducted in all cities (urban and rural) that had at least 5,000 inhabitants. The sampling methods varied among some surveys, the sample sizes ranged between 600 and 54,576 participants, and the collection methods consisted of ‘face-to- face’ or telephone interviews, or self-completed questionnaires (paper or web-based). The response rate was reported in most surveys and ranged between ~46–96%. More details on the survey methodology are in S1 Table.

Birth cohort was calculated by subtracting age at time of survey from the survey year or by using provided birth year. When age of participants was only specified as a multi-year age group, we randomly assigned a single year of age within an age group for the birth cohort calculation, using a penalized composite link model [32], and we checked results were robust to the random assignment. Individual smoking behaviour, specifically whether the participants smoked and if so, the single year of age at smoking initiation was also specified. We defined smoking status as at the time of the survey, with current smokers defined as those who smoked on a daily/regular basis, and former smokers were defined as those who did not smoke daily/regularly at the time of the survey, but reported that they had in the past. We defined ever smokers as the combination of current and former smokers, and never smokers as the remainder, which included those who had smoked on ‘no more than a weekly basis’ in some surveys.

From a total of 398,161 participants, those who did not provide their smoking status, age or sex were excluded (n = 12,351). The final sample (n = 385,810)‬ contained participants born between 1910 and 1989, aged 12 to 99 years at the time of the surveys. From the synthesised data, we constructed longitudinal estimates of the age-specific proportion of current smokers, former smokers and never smokers in the surviving population for each 10-year birth cohort (a supplementary analysis by 5-year cohort was also undertaken). We restricted the results to those based on at least 100 survey respondents and with a relative standard error less than 0.25. Most datasets were supplied with weights (see S1 Table). Data was additionally weighted with post-stratification weights by iterative proportional fitting using ABS population data [33, 34]. We then fitted a binomial, generalised linear model to data for each sex using age and birth-year as continuous covariates to illustrate the line of best fit; while restricting our reporting and discussion to the observed survey proportions. We also plotted the age-specific difference in the proportion of current, former and never smokers for women compared to men for each birth cohort. Because the AGP did not contain information about participants’ past smoking behaviour, the results of the proportion of never and former smoking are based on NHS, RFPS, CCV and NDSHS data only.

Analysis of smoking initiation included surveys that contained information about the age at which daily/regular smoking commenced. These consisted of a representative sample of all Australians from six NHSs conducted between 1989 and 2018, seven NDSHSs conducted triennially between 1998 and 2016, and three RFPSs conducted in 1980, 1983 and 1989.

We defined the age at initiation of smoking as the unweighted self-reported age that smoking commenced daily or regularly (e.g., NDSHS: *“At what age did you first start smoking daily*?*”*, *RFPS*: *“At what age did you start smoking regularly*?*”)*. Ever smokers who were 30 years and younger at the time of survey were excluded from these analyses to minimise bias in the estimated mean age at initiation between the older and younger birth cohorts, since at a whole-of-population level, smoking initiation can continue up to age 30 years. The sample of 98,677 individuals for initiation was used to calculate the sex-specific mean age at initiation for each birth cohort (33,147, 55,859 and 9,671 participants from the NHS, NDSHS and RFPS, respectively) and the proportion of smokers initiating before various ages.

All analyses were performed using R version 3.3.2 [35].

## Results

### Smoking prevalence

**Fig 1:**
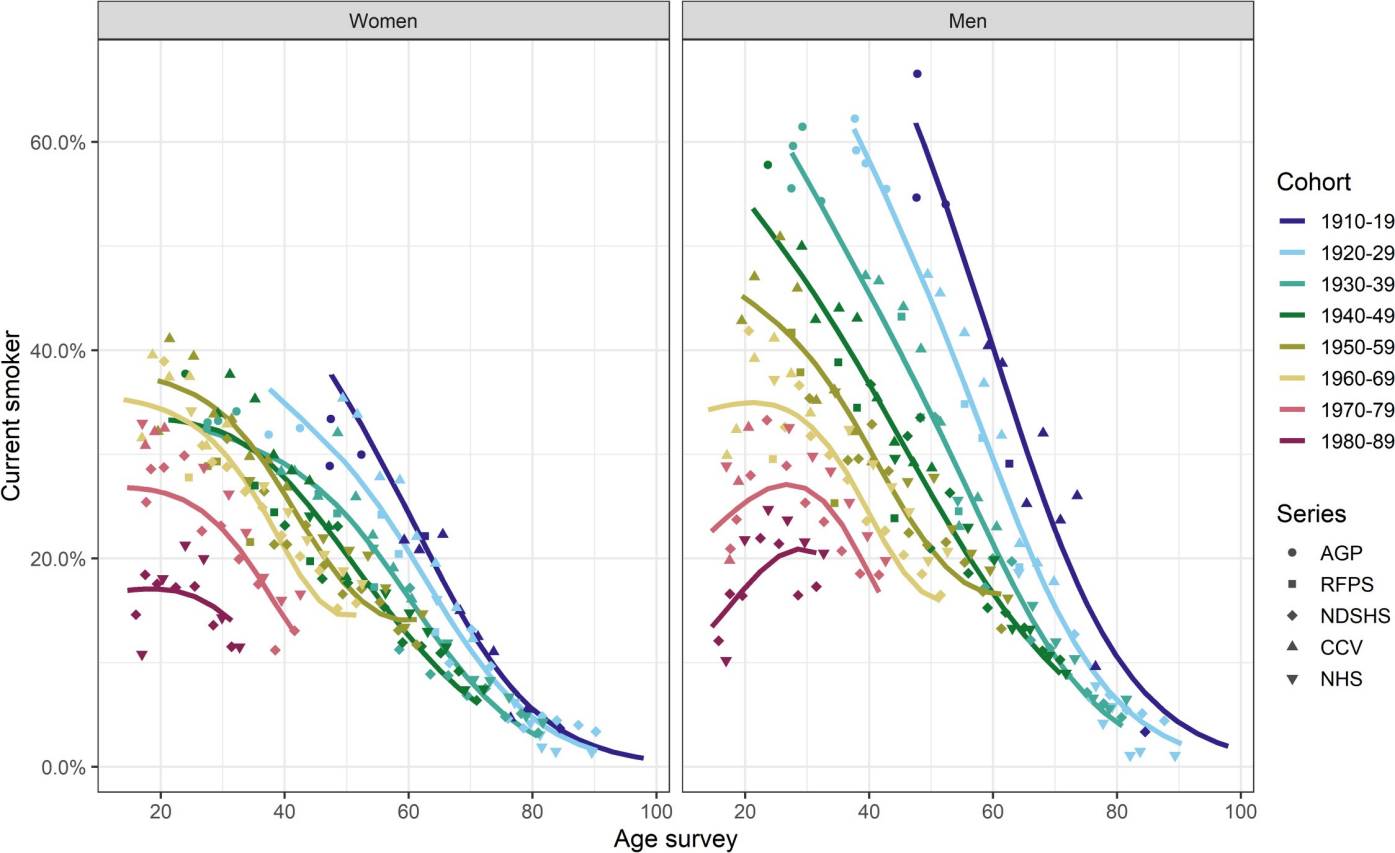
The proportion of current smokers by sex, age and 10-year birth cohort in Australia (Sources: AGP Australian Gallup Polls, CCV Cancer Council Victoria surveys, NDSHS National Drug Strategy Household Surveys, RFPS Risk Factor Prevalence Study and ABS NHS Australian Bureau of Statistics Australian/National Health Surveys).

The proportion of current smokers varied by age and sex across most of the 10-year birth cohorts from 1910 to 1989 (Fig 1). For men, the proportion of current smokers at any age decreased in successive birth cohorts. There was less variation in the proportion of current smokers across birth cohorts of women, especially for those born before 1960. For both men and women, the proportion of current smokers within each of the 1910–19 to 1950–59 birth cohorts decreased with age from the earliest observation (i.e., age ~20–50 years). The subsequent three cohorts (1960-69-1980-89) included participants surveyed before age 20, for whom smoking prevalence increased until the early or mid-twenties and decreased thereafter (Fig 1).

**Fig 2:**
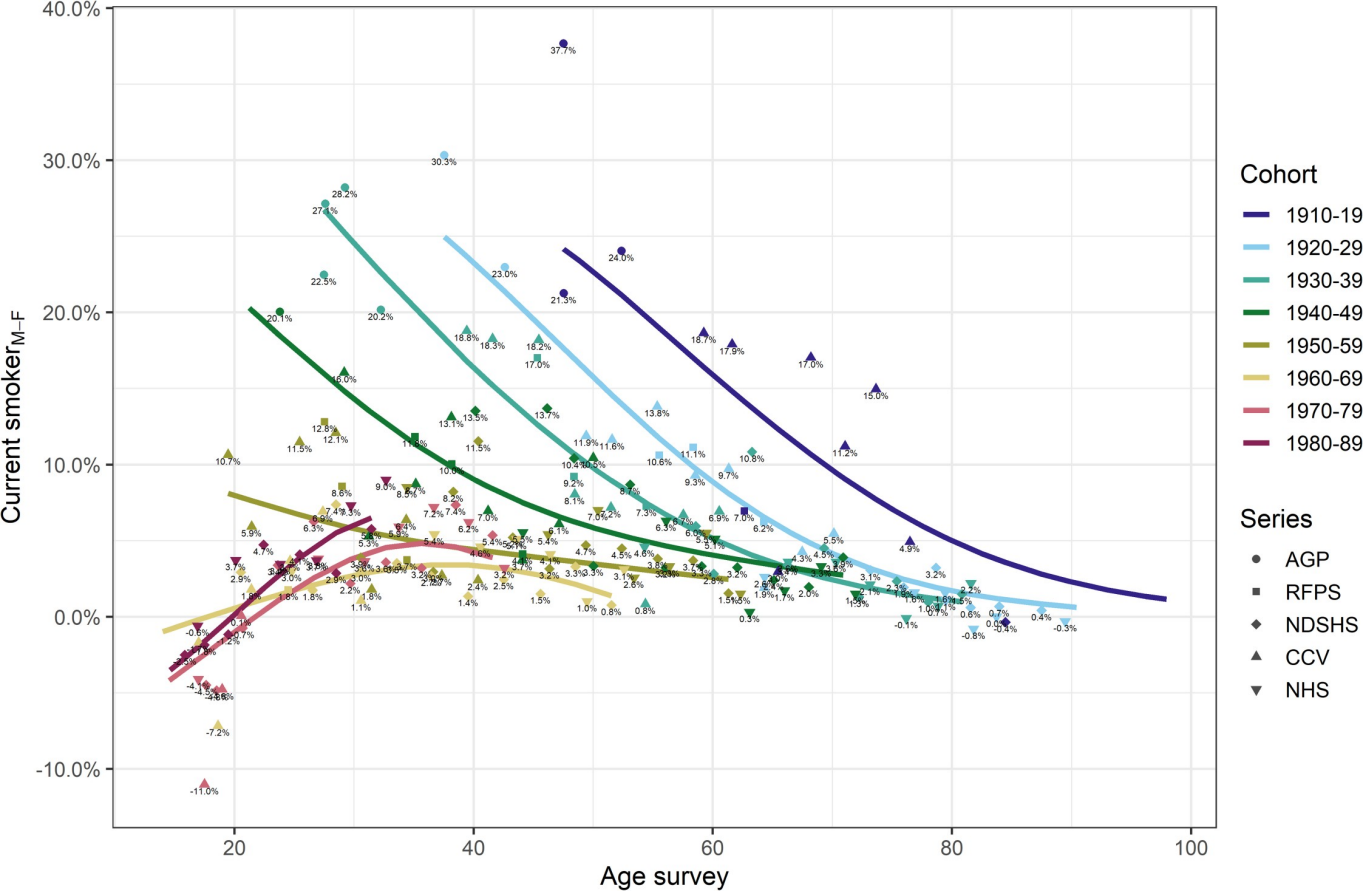
Men-minus-women difference in proportion of current smokers by age and 10-year birth-cohorts in Australia (Sources: AGP Australian Gallup Polls, CCV Cancer Council Victoria surveys, NDSHS National Drug Strategy Household Surveys, RFPS Risk Factor Prevalence Study and ABS NHS Australian Bureau of Statistics Australian/National Health Surveys).

Absolute differences in age-specific proportions of current smokers between men and women for each birth cohort are shown in Fig 2. There was a marked difference in age-specific smoking prevalence among men and women born before 1960–69, where at most, an additional 37.7% of all men smoked compared to the proportion of women. Men and women born in the earliest birth cohort (1910–19) had the largest differences in smoking prevalence compared to other birth cohorts at around the same age, and the difference between men and women generally reduced with each subsequent cohort. For example, at the age of ~40 years (i.e., age when smoking initiation is largely completed and smoking-related mortality does not yet have a major effect on the proportion of ever-smokers within a cohort), up to an additional 30.3% of all men in the 1920–29 cohort smoked compared to women. At that same age, the difference in proportion decreased to 18.8% in the 1930–39 cohort and 13.5% in the 1940–49 cohort (Fig 2), until the proportion did not differ by more than 4.6% between men and women for those born in 1960–1969. However, in the subsequent birth cohort (1970–79), the trend reversed, and proportion of smokers among all men at ~40 years of age was up to 6.2% percentage points higher than among women. Overall, the difference in percentage point of current smokers among men and women declined as the birth cohorts aged. For those born before 1940, the difference in percentage point for current smokers among men and women was at most 2.2% after age 80 years, but this was likely largely driven by smoking-related mortality in men. Generally, the proportion of current smokers was larger in men than women for all cohorts and ages, except for the three latest birth cohorts (1960–69 and 1980–89), where a larger proportion of women than men were current smokers before age 20 years (up to 11.0% difference in percentage points; Fig 2). For these three cohorts, after the age of 20, this pattern reversed, and the proportion of men was slightly larger (up to 3.6% and 7.3% difference in percentage point at age ~30 years).

**Fig 3:**
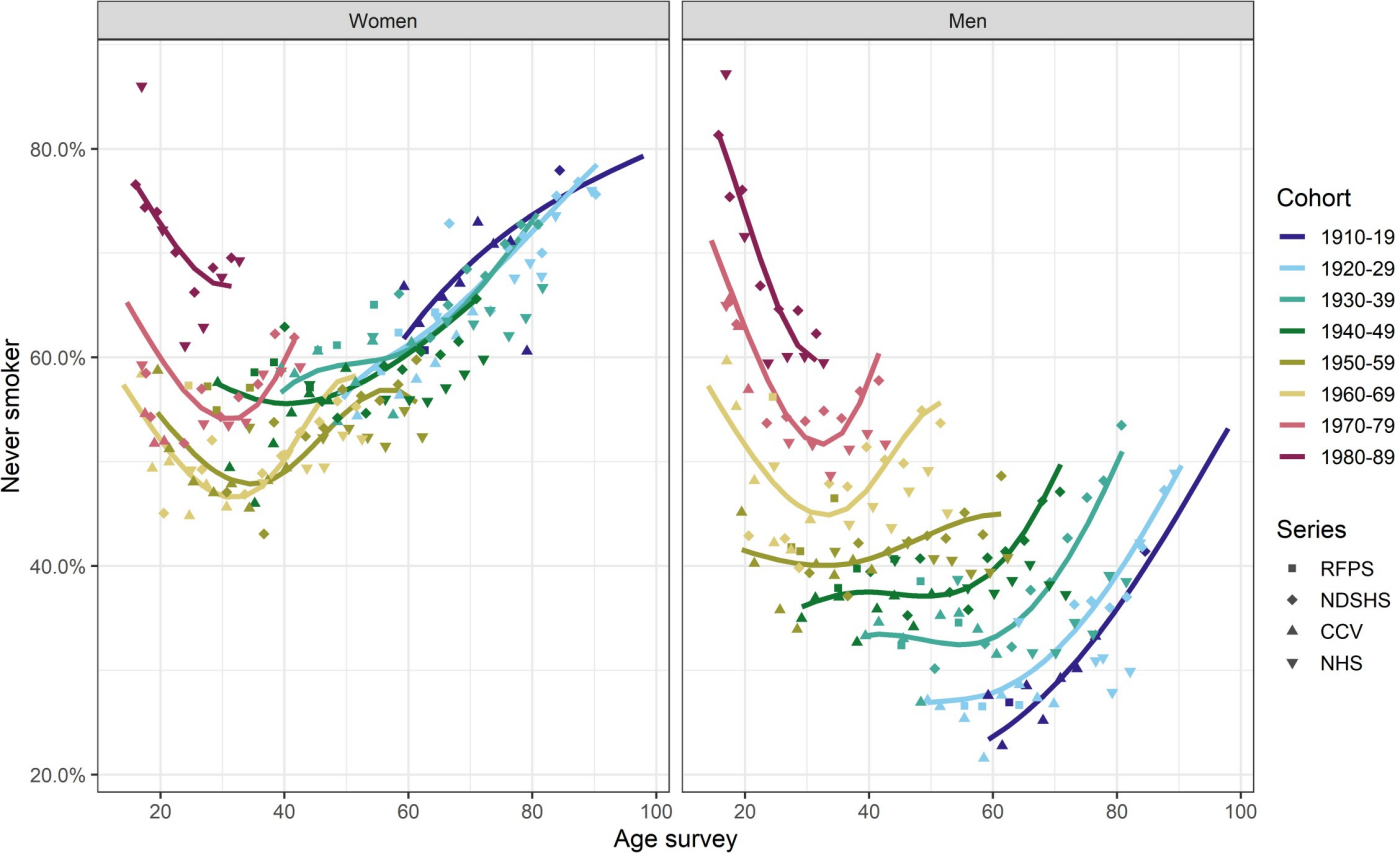
The proportion of never smokers by sex, age and 10-year birth cohort in Australia (Sources: CCV Cancer Council Victoria surveys, NDSHS National Drug Strategy Household Surveys, RFPS Risk Factor Prevalence Study and ABS NHS Australian Bureau of Statistics Australian/National Health Surveys).

**Fig 4:**
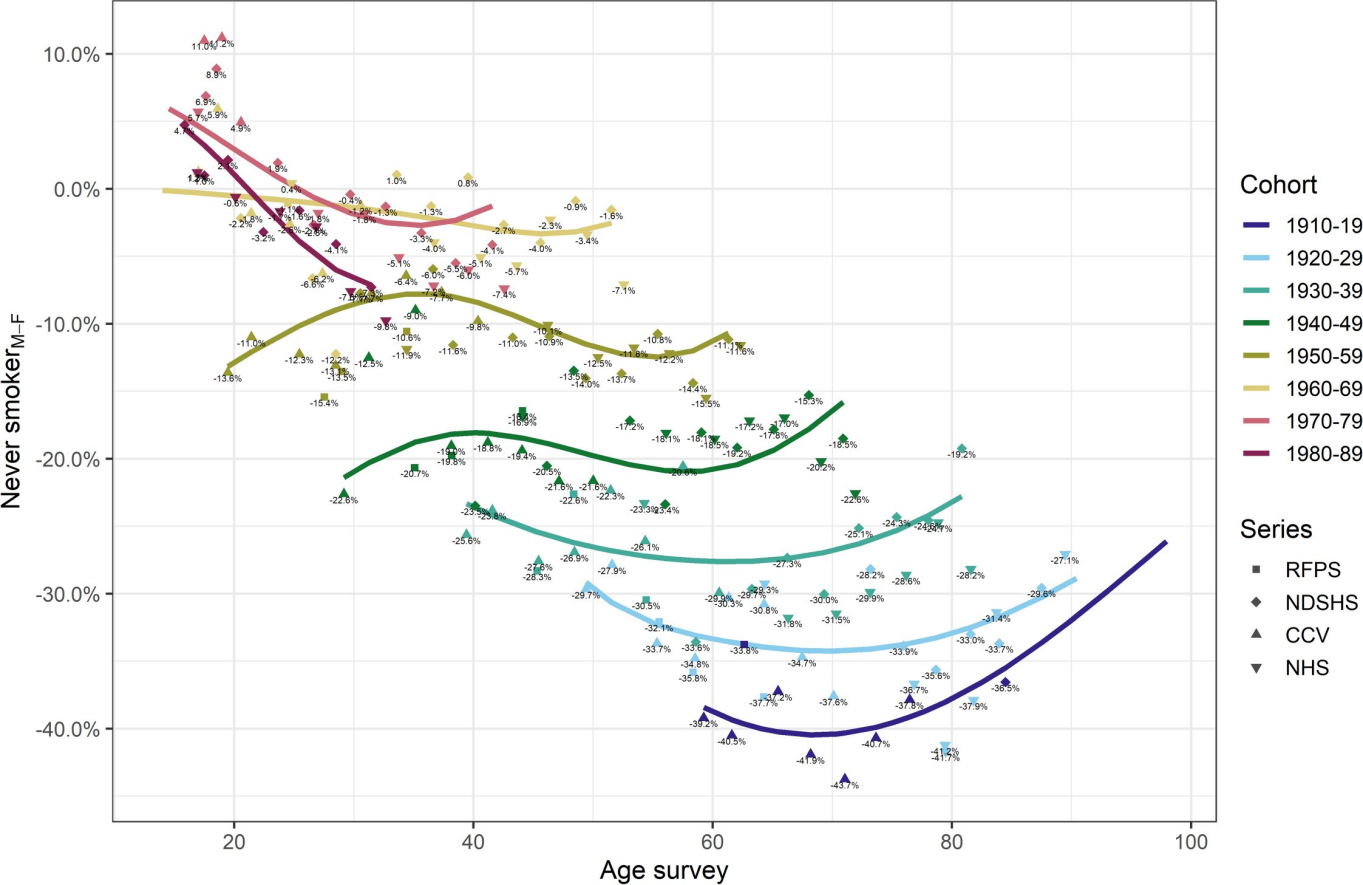
Men-minus-women difference in proportion of never smokers by age and 10-year birth-cohorts in Australia (Sources: CCV Cancer Council Victoria surveys, NDSHS National Drug Strategy Household Surveys, RFPS Risk Factor Prevalence Study and ABS NHS Australian Bureau of Statistics Australian/National Health Surveys).

The estimated age-specific proportion of never smokers in each birth cohort is presented in Fig 3. Among men, the proportion of never smokers, when looking at any fixed age, increased progressively from the earliest to the latest birth cohort. On the other hand, this proportion among women decreased for those born between 1910–19 and 1960–69, as young women initiated smoking at an increasing rate. Also, the proportion of never smokers appeared to increase from around age 30 years onwards in most of the men and all women cohorts born in 1960 and later (Fig 3). The difference in the proportion of never smokers between men and women was generally largest in the earliest cohort of 1910–19 (Fig 4), with, at its most extreme, 43.7% of all women between the age of ~60 and 85 being never smokers compared to men. This gap decreased in the subsequent birth cohorts until there was at most 12.2% difference in percentage points of never smokers between men and women born in 1960 onwards. For those born 1960 and later, there were slightly more never smokers among men than women prior to age 20 years with the percentage points difference in the range of 1.0 to 11.2% (Fig 4).

**Fig 5:**
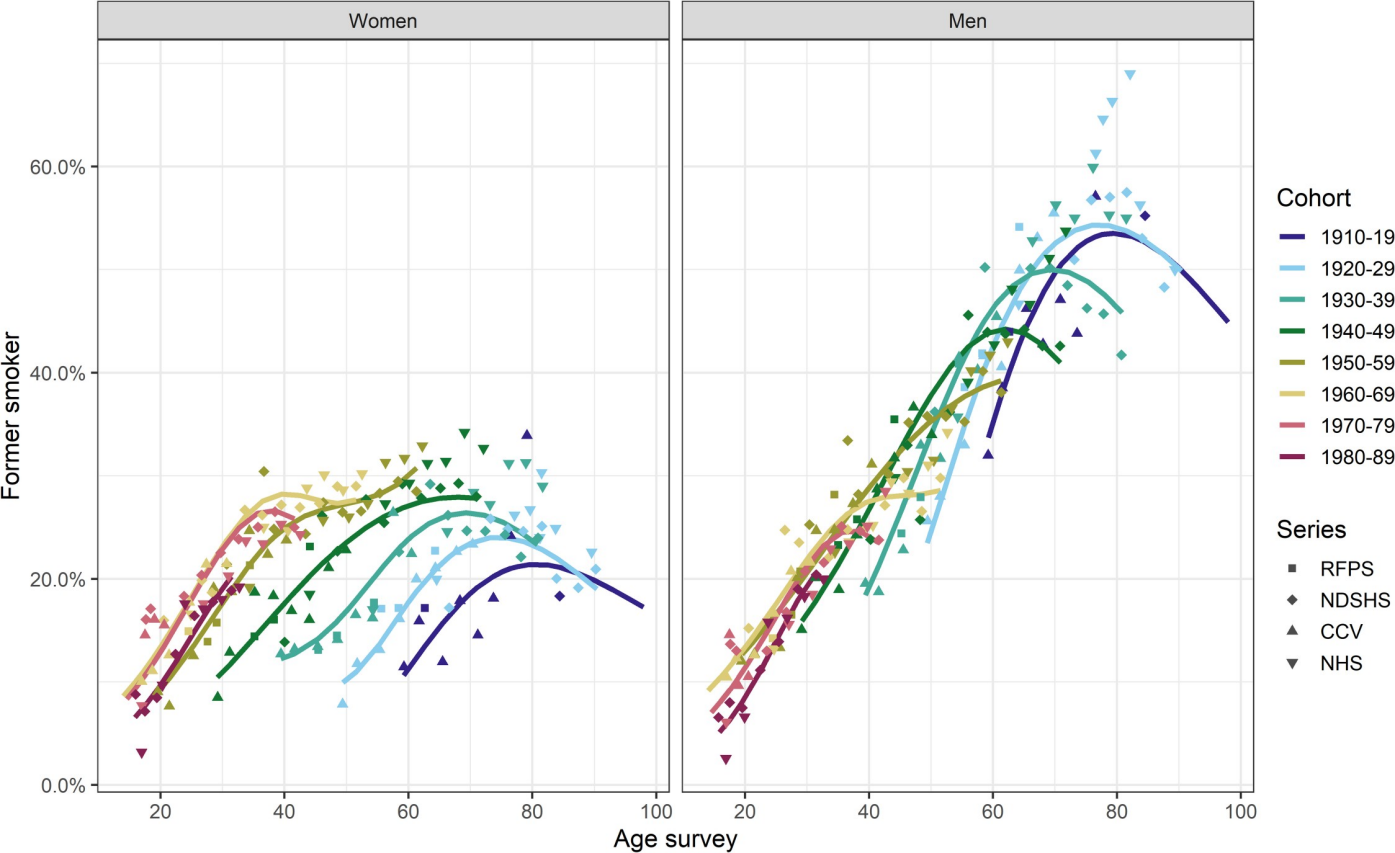
The proportion of former smokers by sex, age and 10-year birth cohort in Australia (Sources: CCV Cancer Council Victoria surveys, NDSHS National Drug Strategy Household Surveys, RFPS Risk Factor Prevalence Study and ABS NHS Australian Bureau of Statistics Australian/National Health Surveys).

**Fig 6:**
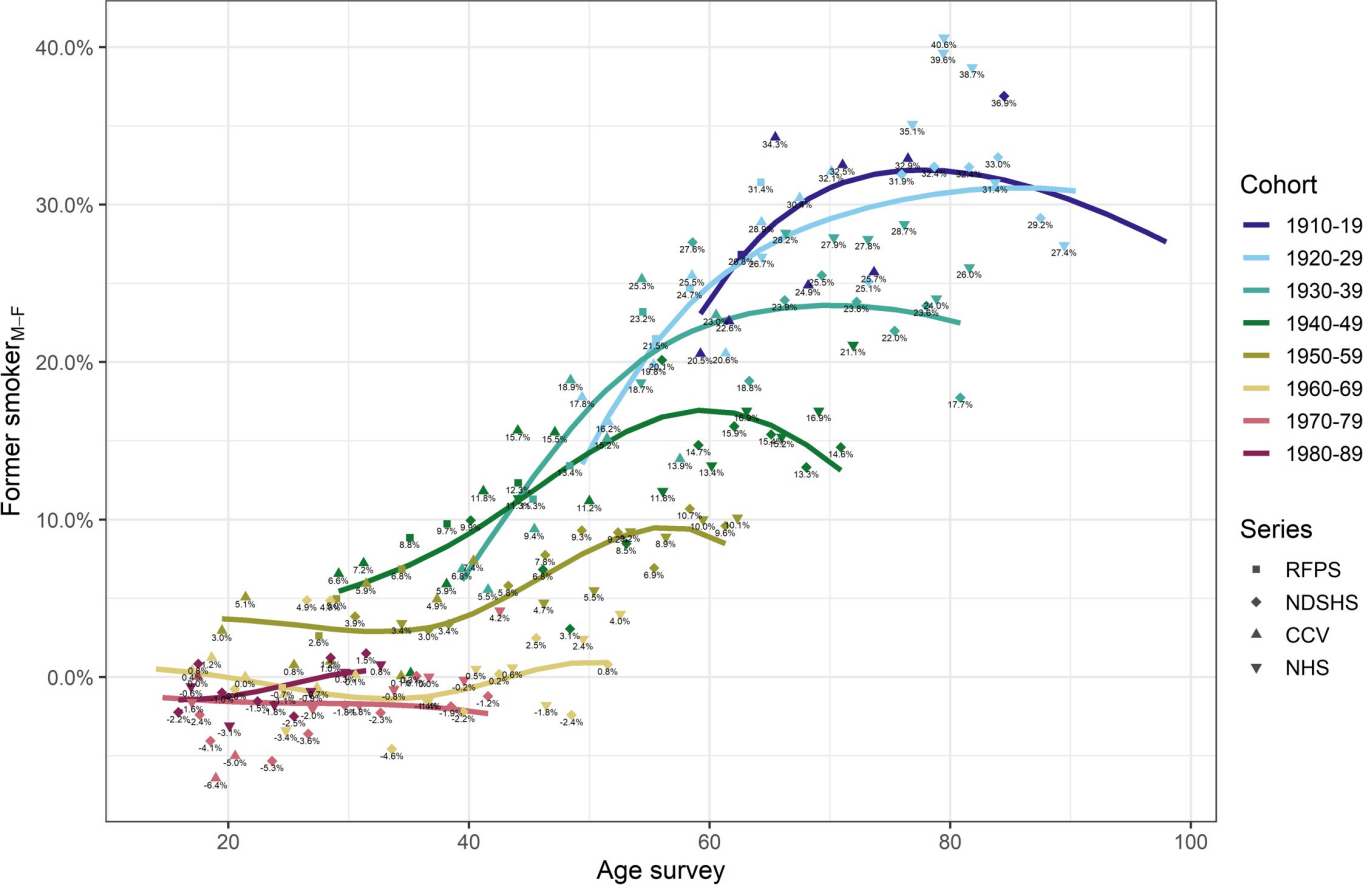
Men-minus-women difference in proportion of former smokers by age and 10-year birth cohorts in Australia (Sources: CCV Cancer Council Victoria surveys, NDSHS National Drug Strategy Household Surveys, RFPS Risk Factor Prevalence Study and ABS NHS Australian Bureau of Statistics Australian/National Health Surveys).

The age-specific proportion of former smokers in each birth cohort increased from the earliest surveyed age for both men and women (Fig 5). The highest proportion of former smokers was among men born in the two earliest birth cohorts (1910–1919 and 1920–29), with up to ~69% of men being former smokers in any survey. Among women, the proportion of former smokers in these two cohorts, was at most ~34.2% (Fig 5) in any survey. Fig 6 shows that the absolute difference in the proportion of former smokers between men and women decreased in subsequent birth cohorts until for those born in 1960–1969 and later, the proportion of former smokers among men and women was similar at all ages, with the difference being no more than 6.4% point across all ages surveyed (i.e., up to age ~40–50 years, Fig 6).

The results of age and sex-specific proportion of current, former and never smokers by 5-year birth cohort showed similar trends and are available in S2–S6 Figs.

### Smoking initiation

The age at smoking initiation differed markedly between men and women in earlier birth cohorts. On average, men born in 1920–29 initiated smoking at the age of 18.2 years, which was approximately 4.4 years earlier than women in this cohort, who initiated smoking at the age of 22.6. While both men and women initiated smoking at increasingly younger ages in the subsequent birth cohorts (i.e., 1930–39 to 1950–59), this was more pronounced among women. The mean age at smoking initiation converged for men and women born in 1960–69, with men initiating smoking at 17.6 and women at 17.8 (Table 1). In the latest two cohorts, 1970–79 and 1980–89, men initiated smoking slightly later than women, with a difference of 0.2 and 0.6 years, respectively.

**Table 1:** Summary statistics of age at smoking initiation by sex and 10-year birth-cohort (excluding those surveyed < = 30 years of age).

	n	Mean	SD	Median	n	Mean	SD	Median
Birth-cohort	Men				Women			
<1919	1,113	19.0	4.7	18	764	26.6	7.6	21
1920–29	5,119	18.2	5.5	18	3,175	22.6	8.8	20
1930–39	8,183	18.0	5.5	18	5,254	21.5	7.9	20
1940–49	13,214	17.6	4.7	17	10,281	19.8	6.2	18
1950–59	13,742	17.4	4.5	17	12,514	18.6	5.4	18
1960–69	10,076	17.6	4.5	17	11,827	17.8	4.5	17
1970–79	6,346	17.9	4.3	18	7,530	17.7	4.0	17
1980–89	1,267	18.0	3.6	18	1,340	17.4	3.5	17

Table 2 shows the change in proportion of men and women who initiated smoking by specific age thresholds. A larger proportion of men than women born before 1960–69 initiated smoking early in their lives, with the greatest difference for those who initiated smoking by the age of 20. For example, 23.5% of men and only 9.2% of women born in 1920–29 initiated smoking before the age of 16. Although this proportion increased for both men and women born in the subsequent cohorts, the increase was distinctly higher among women. Among those born in 1930–39, only one woman for every two and half men initiated smoking before the age of 16 (27.6% of men vs 10.0% of women). This increased to one woman for every two men for those born in 1940–49 (28.1% of men vs 13.9% of women). Finally, for those born in 1970–79, the proportion of men and women who initiated smoking before the age 16 was approximately the same, with a difference of only 1.1%.The proportion of both men and women initiating before the age of 16 peaked in those born in 1960–69, with 30.7% of men and 27.7% women (i.e., approximately one out of every three of all ever-smokers in that cohort) initiating daily/regular smoking by that age. There was a higher proportion of women than men initiating smoking before the age of 16 in the 1980–89 birth cohort, with approximately 7.4% more women than men initiating at this age.

**Table 2:** Percentage at smoking initiation among ever smokers by age, sex and 10-year birth-cohort calculated from empirical distributions (excluding those surveyed < = 30 year of age).

	Aged < = 15 (%)		Aged < = 20 (%)		Aged < = 25 (%)		Aged < = 30 (%)		Aged < = 35 (%)		Aged < = 40 (%)	
	M	F	M	F	M	F	M	F	M	F	M	F
1910–19	22.9	8.4	72.3	51.7	92.0	73.0	97.6	89.1	99.2	94.8	99.2	100.0
1920–29	23.5	9.2	83.2	58.9	96.3	81.3	98.9	91.2	99.6	95.6	99.6	100.0
1930–39	27.6	10.0	83.0	62.5	95.7	84.2	98.6	93.2	99.3	97.0	99.3	100.0
1940–49	28.1	13.9	86.5	74.0	96.8	90.6	99.0	96.3	99.6	98.7	99.6	100.0
1950–59	29.6	19.4	87.0	82.4	96.6	94.6	99.0	98.2	99.6	99.2	99.6	100.0
1960–69	30.7	27.7	85.0	86.0	95.3	95.5	98.3	98.1	99.3	99.1	99.3	100.0
1970–79	26.2	27.3	81.4	83.8	95.2	95.9	98.5	98.7	99.8	99.6	99.8	100.0
1980–89	21.4	28.8	82.7	87.5	96.1	96.9	99.5	99.6	100.0	100.0	-	-

A larger proportion of women born in the earlier cohorts initiated smoking at later ages than men (Table 2). For example, 18.7% of women born in 1920–29 initiated smoking after the age of 25, while a maximum of 4.8% of men initiated after that age in this and consequent birth cohorts. For those born in 1960–69 and later, less than 2% of both men and women initiated smoking after the age of 30.

S2–S5 Tables present 5-year birth-cohort-specific trends in smoking initiation for men and women.

## Discussion

There have been marked differences in the patterns of smoking prevalence and initiation between birth cohorts of men and women over the past 80 years in Australia, with the largest differences occurring in the earliest cohorts. Among men, smoking prevalence decreased gradually between successive cohorts as fewer and fewer men initiated smoking and those who had started smoking quit at increasingly younger ages. The mean age at initiation among men who had smoked daily/regularly varied little between birth cohorts, with only a small shift in the 1980–89 birth cohort where men initiated smoking marginally later than in earlier cohorts. Far fewer women than men smoked in the earlier cohorts, and those that did smoke started at a later age than men on average. The proportion of smokers among Australian women born in the first half of the century increased, and their mean age at initiation decreased with each successive birth cohort until the smoking patterns of women born in the 1960s closely resembled those of men. Among both men and women born after the 1960s, the smoking prevalence decreased in a similar fashion in each consecutive birth cohort. Overall, smoking prevalence was lower among women than among men, although there was a larger proportion of current smokers among women younger than 20 years in the most recent cohorts, and among some older women in the earlier cohorts.

Our results were found to be broadly consistent with patterns in other, smaller, Australian studies. Specifically, two prior Australian studies utilised similar methods to our work, and compared age-specific smoking prevalence across 10-year birth cohorts. These studies were based on either one (i.e., the Household, Income and Labour Dynamics in Australia) [36] or two (NDSHS 2001 and 2004) [23] national surveys. Our results, using a larger sample across many surveys, enhance and extend the results of these prior analyses. The report by Lillard (2010) also compared birth cohort trends to those observed in the US and the UK, finding that the prevalence of smoking among women born in Australia peaked later than those born overseas (1960s vs. 1940s in the US and the UK), whereas the trends among men were roughly the same [36]. The observed patterns of prevalence among current and former smokers in our study closely follow modelled trends of current and former smokers in the US, except for the 1980–89 birth cohort, where prevalence in both current and former smokers is somewhat lower among the Australian survey participants [37].

The patterns of cohort-specific smoking prevalence and initiation that we have constructed using cross-sectional survey data were also similar to those found in a longitudinal study of a small cohort in Busselton, Western Australia [38]. The study, in which the same individuals were re-surveyed over time, observed that the proportion of never smokers born between 1895 and 1955 increased among men and decreased among women until it became similar (i.e., 53% and 56%) for both men and women born in the late 1950s. Similar to our results, the decrease in the age of mean initiation was smaller for men than women. These results suggest that estimates of life-course smoking behaviours reconstructed from population-based cross-sectional data yield a reasonable estimate of population-based longitudinal trends, which are currently unavailable. By synthesising data on more than 380,000 people from many surveys, we were able to characterise behaviour for eight 10-year birth cohorts at a population level.

Our study observed an increase with age in the proportion of men and women reporting that they were never smokers within the same birth cohort (i.e., in more recent birth cohorts after the age of 30). The drivers of this increase in never-smoker responses could theoretically include any combination of the effects of out-migration or immigration, mortality, recall bias and recanting (where survey participants switch smoking categories later in their lives). Recanting, which cannot be measured here, has been observed in previous longitudinal studies which found that up to 20% of former smokers (re)defined themselves as never-smoker in later surveys [23, 38].

Studies of birth cohorts have been important for understanding how social, economic, political, informational, regulatory and technological events underpin historical and contemporary patterns of tobacco use. In Australia, smoking before World War I (WWI) was prevalent predominantly among men, since smoking among women was not socially acceptable [39]. The use of tobacco among men increased dramatically during WWI when cigarettes were rationed to serving men by the Australian Government, but also to those women who served alongside them [22, 40]. Tobacco was thought to reduce stress and increase comradery [40]. In the 1920s, soon after the first cohort of our study was born, social norms changed with more women participating in the workforce, gaining financial independence and spending more time outside of their home due to technological developments of domestic tools. Smoking became more socially acceptable for women [41]. The tobacco industry exploited the changing economic and social role of women and intensified marketing of cigarettes towards women [42]. The first (oldest) birth cohort of our study reached adolescence and young adulthood in the early 1930s and during WWII. During this time women gained increasingly more independence by participation in the workforce while men (and some women) who served in the war were once more supplied with cigarettes. By the end of the war, 25% of women and 75% of men smoked [39, 43].

After WWII, smoking prevalence decreased among men but increased among women who continued to gain rights, freedom and privileges and with them, more social and financial power [22]. Tobacco advertising intensively targeted women and portrayed attractive and famous women promising slimness, social success, and freedom [44]. The purchase of cigarettes also provided women with discounts to other domestic staples such as milk or laundry detergents [42–44]. In 1961, oral contraceptives became legally available in Australia, increasing the autonomy of women in society [45]. The prevalence of smoking peaked in our study for women born in the 1960s. Growing up, these women would have witnessed the previous generation of women smoking at high numbers, having themselves comparatively high social and financial freedom, with marketing of tobacco yet barely regulated and health campaigns still relatively limited. Although the US Surgeon General’s report published in 1964 highlighted the relationship between tobacco use among men and cancer and a range of other diseases [46], the public health policy response was slow. Significant tobacco control measures in Australia were delayed until the first pack warning in 1973, and the phasing out of broadcast tobacco advertising (later followed by print media) from 1976–80 followed by a range of other reforms and initiatives, associated with the gradual, long-term decline in smoking among both men and women to the current 11.0% of daily smokers aged 14 and over [4, 47, 48].

Our study reports the largest and most informative new evidence about the life-course smoking patterns in Australia to date. As with all retrospective surveys based on self-reported questions, it is limited by response and recall biases, thus potentially underestimating the proportion of smokers and former smokers. Even though most of the surveys used in our study are nationally representative and represent the best data of smoking behaviour available to us, some surveys did not report a response rate or had less than optimal rates of response (i.e., <70%), or in some early cases were based on a small sample. A low response rate may introduce bias in our results when answers of respondents are systematically different from those who did not participate, although this can, under strict conditions, be partially corrected with weighting procedures we used. The small sample size in early surveys affects the precision of our estimates for the earlier ages observed of those born in the first half of the century, and therefore the corresponding results should be interpreted with caution.

Another limitation is that retrospective data do not measure the past smoking behaviour of all participants, because smokers are more likely to die than non-smokers [49]. Thus, due to this differential mortality, our results for age at smoking initiation (i.e., mean age, and proportion initiated by key ages) for the birth cohorts that were solely surveyed at older ages (i.e., responses captured in the earliest surveys) are likely to be biased, whereby surviving smokers in birth cohorts only captured at ages 50–60 years or older may have started smoking later in life. This effect could be larger among men due to their higher consumption of cigarettes leading to a higher probability of dying before being surveyed. For birth cohorts that were predominantly surveyed before age 50 years, the observed age at initiation results are comparatively more reliable estimates of the true mean age at initiation.

Our findings are useful given that large-scale representative ongoing longitudinal studies of tobacco exposure which follow specific birth cohorts have not been reported in Australia. Previous birth-cohort studies in Australia were constructed on either a smaller number of surveys [23, 36, 50], were not analysed by sex [14], or were based on a small sample of survey participants [38]. Our study enhanced the current knowledge by combining multiple national surveys, which allowed us to build 10-year (and in supplementary analysis, 5-year) birth cohorts for both sexes that provided historical trends in smoking prevalence and initiation. Our findings will be critical to the ongoing development of population-based macro- and microsimulation models of tobacco-related disease and mortality. Crucial to the development of such models is an understanding of age-period-cohort level exposure to smoking in the population. In general, comparing life-course smoking behaviours across birth cohorts can present a more dynamic picture of smoking patterns and can be mapped contemporaneously to tobacco control initiatives as well as social and political determinants of health at particular time points. Accurately quantifying the impact of tobacco control measures on smoking behaviours by age, sex and calendar year is important for informing ongoing investment in reducing tobacco-related harm. Given the 20–30 year lag between population-level smoking exposure patterns and its impact on rates of disease (e.g., lung cancer [51]), estimations of historical longitudinal smoking patterns can also be used to predict patterns of disease outcomes in the future.

## Supporting information

S1 Fig The proportion of current smokers by sex and 5-year birth cohort in Australia (AGP Australian Gallup Polls, CCV Cancer Council Victoria survey, NDSHS National Drug Strategy Household Survey, RFPS Risk Factor Prevalence Study). Australian/National Health Survey was excluded due to small numbers in some birth cohorts.
(DOCX)

S2 Fig Men-minus-women difference in proportion of current smokers by age and 5-year birth-cohorts in Australia (Sources: AGP Australian Gallup Polls, CCV Cancer Council Victoria surveys, NDSHS National Drug Strategy Household Surveys, RFPS Risk Factor Prevalence Study). Australian/National Health Survey was excluded due to small numbers in some birth cohorts.
(DOCX)

S3 Fig The proportion of never smokers by sex, age and 5-year birth cohort in Australia (Sources: CCV Cancer Council Victoria surveys, NDSHS National Drug Strategy Household Surveys, RFPS Risk Factor Prevalence Study). Australian/National Health Survey was excluded due to small numbers in some birth cohorts.
(DOCX)

S4 Fig Men-minus-women difference in proportion of never smokers by age and 5-year birth-cohorts in Australia (Sources: CCV Cancer Council Victoria surveys, NDSHS National Drug Strategy Household Surveys, RFPS Risk Factor Prevalence Study). Australian/National Health Survey was excluded due to small numbers in some birth cohorts.
(DOCX)

S5 Fig The proportion of former smokers by sex, age and 5-year birth cohort in Australia (Sources: CCV Cancer Council Victoria surveys, NDSHS National Drug Strategy Household Surveys, RFPS Risk Factor Prevalence Study). Australian/National Health Survey was excluded due to small numbers in some birth cohorts.
(DOCX)

S6 Fig Men-minus-women difference in proportion of former smokers by age and 5-year birth cohort (CCV Cancer Council Victoria Survey, NDSHS National Drug Strategy Household Survey, RFPS Risk Factor Prevalence Study/Survey). Australian/National Health Survey was excluded due to small numbers in some birth cohorts.
(DOCX)

S1 Table Summary of individual surveys use in our study (Note: VDSHS 1993 and 1995 surveys were combined with NCADASIS 1993 and NDSHS 1995, respectively, to create 33 surveys). ADA Australian Data Archive: https://dataverse.ada.edu.au.
(DOCX)

S2 Table Mean age of smoking initiation among ever-smokers by sex and survey series for 10-year birth-cohort in Australia (excluding those surveyed at 30 years and younger). NHS Australian/National Health Survey NDSHS: National Drug Strategy Household Survey RFPS: Risk Factor Prevalence Study/Survey.
(DOCX)

S3 Table Smoking initiation by age, sex and 10-year birth-cohort calculated from empirical distributions in Australia (excluding those surveyed at 30 years and younger). NHS Australian/National Health Survey NDSHS: National Drug Strategy Household Survey RFPS: Risk Factor Prevalence Study/Survey.
(DOCX)

S4 Table Mean age of smoking initiation among ever-smokers by sex and 5-year birth-cohort (excluding those surveyed at 30 years and younger). NHS Australian/National Health Survey NDSHS: National Drug Strategy Household Survey RFPS: Risk Factor Prevalence Study/Survey.
(DOCX)

S5 Table Smoking initiation by sex, age and 5-year birth-cohort calculated from empirical distributions (excluding those surveyed at 30 years and younger). NDSHS: National Drug Strategy Household Survey RFPS: Risk Factor Prevalence Study/Survey *Australian/National Health Survey was excluded due to small numbers in some birth cohorts.
(DOCX)
