# Supplementary material for: Birth-cohort estimates of smoking initiation and prevalence in 20th century Australia: Synthesis of data from 33 surveys and 385,810 participants
Source: PLoS One. 2021 May 21;16(5):e0250824. doi: 10.1371/journal.pone.0250824 (PMC8139520; doi:10.1371/journal.pone.0250824)
Supplement: S3 Fig — Australian/National Health Survey was excluded due to small numbers in some birth cohorts. (DOCX) [file pone.0250824.s003.docx]

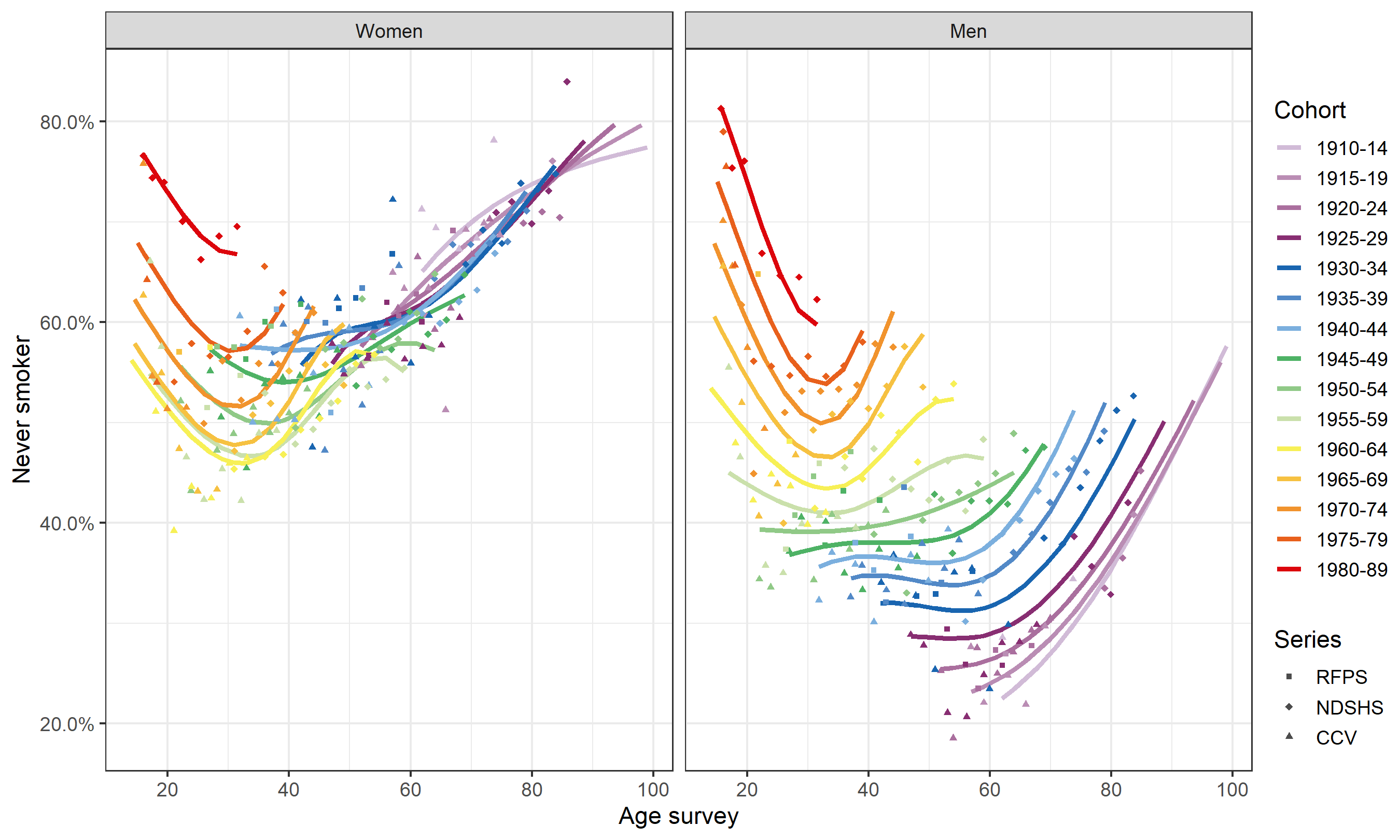
S3 Fig. The proportion of never smokers by sex, age and 5-year birth cohort in Australia (Sources: CCV Cancer Council Victoria surveys, NDSHS National Drug Strategy Household Surveys, RFPS Risk Factor Prevalence Study). Australian/National Health Survey was excluded due to small numbers in some birth cohorts.
