# Supplementary material for: Birth-cohort estimates of smoking initiation and prevalence in 20th century Australia: Synthesis of data from 33 surveys and 385,810 participants
Source: PLoS One. 2021 May 21;16(5):e0250824. doi: 10.1371/journal.pone.0250824 (PMC8139520; doi:10.1371/journal.pone.0250824)
Supplement: S1 Table — ADA Australian Data Archive: https://dataverse.ada.edu.au. (DOCX) [file pone.0250824.s007.docx]

| S1 Table. Summary of individual surveys use in our study (Note: VDSHS 1993 and 1995 surveys were combined with NCADASIS 1993 and NDSHS 1995, respectively, to create 33 surveys). ADA Australian Data Archive: https://dataverse.ada.edu.au | | | | | | | |
| --- | --- | --- | --- | --- | --- | --- | --- |
| **ADA ID/ Survey name/URL address** | **Year** | **Sample characteristics** | **Collection method** | **Response rate** | **Weighting** | **Short description** | **Source** |
| 00093-Australian Gallup Polls Survey (AGPS), Survey 158 | 1962 | n=2,086  Aged 21+ throughout Australia. | A face-to-face interview | Not available | Unknown | Sampling method: area-cluster sample with sex quota.  Time method: one-time cross-sectional study - partial replication (Omnibus survey conducted seven or eight times annually).  Data collector: Roy Morgan | ADA  https://dataverse.ada.edu.au/dataset.xhtml?persistentId=doi:10.26193/HMLYBV |
| 00308, Australian Gallup Polls Survey (AGPS), Survey 160 | 1962 | n=1,615  Aged 21+ throughout Australia. | A face-to-face interview | Not available | Unknown | Sampling method: area-cluster sample with sex quota.  Time method: one-time cross-sectional study - partial replication (Omnibus survey conducted seven or eight times annually).  Data collector: Roy Morgan | ADA  https://dataverse.ada.edu.au/dataset.xhtml?persistentId=doi:10.26193/67CUQK |
| 00096 - Australian Gallup Polls Survey (AGPS) | 1964 | n=1,375  Aged 21+ throughout Australia. | Personal interview | Not available | YES  Weighting: weighting criteria unknown. | Sampling method: area-cluster sample with sex quota.  Time method: cross-sectional (one-time) study - partial replication Omnibus survey conducted eight times annually.  Data collector: Roy Morgan | ADA  https://dataverse.ada.edu.au/dataset.xhtml?persistentId=doi:10.26193/ZBHA9M  http://www.ada.anu.edu.au/social-science/00096 |
| 00327-Australian Gallup Polls Survey (AGPS) | 1967 | n=1,823  Aged 21+ throughout Australia. | Personal interview | Not available | NO | The responses are unweighted.  Time method: cross-sectional (one-time) study partial replication Omnibus survey conducted seven or eight times annually.  Sampling procedure: area-cluster sample with sex quota.  Data collector: Roy Morgan | ADA  https://legacy.ada.edu.au/social-science/00327 |
| 00049 - Tobacco smoking patterns in Australia (TSPA) | 1974 | n=6,625  Australians aged 14+. | Personal interview | Non-response:  40% | NO | Authors: Gray, Nigel (Anti-Cancer Council of Victoria) Hill, David (Anti-Cancer Council of Victoria)  Data Collector : Roy Morgan Research Centre | ADA  https://dataverse.ada.edu.au/dataset.xhtml?persistentId=doi:10.26193/WEKJGJ  Pierce (1989). International Comparisons of Trends in Cigarette Smoking Prevalence. American Journal of Public Health, Vol. 79, No.2, Pg. 152-157. |
| Cancer Council Victoria (CCV) Survey | 1976 | n=3,873  Aged 16+. | Personal interview? | Non-response:  40% | Unknown | A cross-sectional study. | Patterns of Tobacco Smoking in Australia  N.J. Gray and D. J. Hill  Med J Aust, 1977,2: 327-328  Pierce (1989). International Comparisons of Trends in Cigarette Smoking Prevalence. American Journal of Public Health, Vol. 79, No.2, Pg. 152-157. |
| 00048 – Patterns of tobacco smoking in Australia:3  (PTSA) | 1980 | n=4,476  Australians aged 14+. | Personal interview | Non-response:  40% | NO | Questions on smoking behaviour were embedded in a multi-faceted “omnibus” survey conducted by Roy Morgan Research Centre.  Primary investigators: Gray, N. Hill, D.  Related publications: Hill, D. J. and N. J. Gray. Patterns of tobacco smoking in Australia. Medical Journal of Australia, 1982, pp. 23-25.  Data collector: Roy Morgan Research Centre | ADA  https://dataverse.ada.edu.au/dataset.xhtml?persistentId=doi:10.26193/5BEQHJ  https://legacy.ada.edu.au/social-science/00048  Pierce (1989). International Comparisons of Trends in Cigarette Smoking Prevalence. American Journal of Public Health, Vol. 79, No.2, Pg. 152-157. |
| 00122 – Risk Factor Prevalence Study (RFPS) | 1980 | n=5,603  Aged 25-64, residents of Australian capital cities listed on state electoral rolls. | A self-completed questionnaire (distribute, collect) accompanied by a clinical test | 75.9% | YES | Sampling procedure: Multi-stage sample Seven ‘catchment’ areas from which the sample was to be drawn were selected: Sydney North, Sydney South, Melbourne, Brisbane, Adelaide, Perth and Hobart. The population of each area was stratified according to age, sex and electoral division and a representative sample of 1200 persons per area was selected. Letters of invitation were sent to the people selected inviting them to attend the centre in their area. A questionnaire was completed in the presence of the centre’s clerk who then coded it while the respondent was available to clarify any inconsistencies. A nursing sister then performed a medical examination and collected a blood sample.  Author: National Heart Foundation of Australia | ADA  https://dataverse.ada.edu.au/dataset.xhtml?persistentId=doi:10.26193/BYE1RE  https://legacy.ada.edu.au/social-science/00122  Hodge (1994). Risk factors in Australians: National Heart Foundation’s Risk Factor Prevalence Study, 1980. [Australian and New Zealand Journal of Medicine](https://onlinelibrary.wiley.com/journal/14455994a), [Vol. 14, Iss 4](https://onlinelibrary.wiley.com/toc/14455994a/1984/14/4), Pg. 395-399. |
| 00339 – Smoking survey, Australia (SSA) | 1983 | n=5,739  Persons aged 16+ years. | Personal interview | Non-response:  40% | YES  Weighting: weighting criteria the use of age, sex and locality in relation to known frequencies to obtain representativeness weighting procedures 88 sex-age-locality groups were weighted to their correct proportions for the latest estimates available for the population aged 14 years and over. The groups are comprised of all combinations of two sexes, 4 ages (14-24, 25-34, 35-49, 50+), and 11 localities (the 5 mainland capitals, the 5 remaining mainland regions, and Tasmania). | Sampling procedure: An area-cluster sample. The interviews were conducted at weekends in five weekly waves, within each of 110 electoral subdivisions chosen at random within 13 strata based upon the six states and the ACT and NT split into urban and rural categories. Within each of these subdivisions one elector’s name was selected at random. The residence adjoining this elector’s was the first contact dwelling and further adjoining dwellings were approached until the quota of ten interviews was filled. Replacements accounted for 39 per cent of the final sample, one fifth being replacements of refusals and one fifth being replacements of unattended households.  Respondents were interviewed in their homes. One interview per household was conducted and interviewers adhered to a respondent selection key, rotating between various age/sex combinations present at the time of calling. Questions about smoking habits and related issues were embedded in an omnibus survey serving various clients of the market research company. It is these smoking questions only that are included in this data set.  Data collector: Roy Morgan Research Centre | ADA  https://dataverse.ada.edu.au/dataset.xhtml?persistentId=doi:10.26193/RRCTHD  http://legacy.ada.edu.au/social-science/00339  Hill D and N Gray (1984). Australian patterns of tobacco smoking and related health beliefs in 1983. Community Health Studies, Vol. 8, Iss. 3, Pg. 307-316.  Pierce (1989). International Comparisons of Trends in Cigarette Smoking Prevalence. American Journal of Public Health, Vol. 79, No.2, Pg. 152-157. |
| 00414 – Risk factor prevalence study (RFPS) | 1983 | n=7,615  Persons aged 25-64 years. | A self-completed questionnaire (distribute, collect) accompanied by a clinical test | 74.7%  (Note: this is a combined response rate across 1980, 1983 and 1989 RFPS) (Please see: AIHW 2003. Indicators of health risk factors: the AIHW view. AIHW Cat. No. PHE 47. Canberra: AIHW). | YES | Sampling procedure: multi-stage sample with quota. The sample was selected from seven catchment areas – Sydney north, Sydney south, Melbourne, Brisbane, Adelaide, Perth and Hobart – using the Commonwealth electoral roll. All electoral divisions and sub-divisions within a radius of 16km of a National Heart Foundation centre were included in the study. Initial sample sizes of 1500 in each catchment area were required, with the exception of Perth where a sample size of 2400 was needed, to satisfy requirements for its participation in the World Health Organisation’s MONICA Project. The population of each catchment area was stratified according to age, sex and electoral division, and a representative sample was selected, following the quotas mentioned above.  Letters of invitation giving a specific appointment were posted about two weeks in advance of the appointment date. Upon arrival at a centre, respondents would be handed a questionnaire to be completed, and would then be referred to a nursing sister for physical and blood pressure measurements. After the physical examination respondents were also interviewed by a dietician. | ADA  https://dataverse.ada.edu.au/dataset.xhtml?persistentId=doi:10.26193/RRCTHD  https://legacy.ada.edu.au/social-science/00122 |
| 00405 – Social issues in Australia (SIA) | 1985 | n=2,791  Persons aged 14+ living in urban centres with a population of 5,000 or more persons | A face-to-face interview and self-completion questionnaire |  | NO | Stratified area-cluster sample with age-sex quotas.  Urban centres with a population of 5000 or more persons were stratified according to 15 regions defined by the six State capital cities and the remainder of each State, Darwin and the remainder of the Northern Territory, and Canberra. Each State was to return a quota of 400 interviews and each Territory 200 interviews. | ADA  https://dataverse.ada.edu.au/dataset.xhtml?persistentId=doi:10.26193/LIZHRW  http://legacy.ada.edu.au/social-science/00405 |
| Cancer Council Victoria (CCV) Survey | 1986 | n=9,440  Persons aged 16+ years living in Australia. | An interview at home | Non-response:  40% | Unknown. | A random sample survey was conducted in 11 electoral subdivision, which were chosen at random with 13 strata that were based upon 5 states and the Australian Capital Territory and Northern Territory, as split in rural and urban categories. A part of an omnibus survey.  Data collector: a large market-research company – a name was not stated. | Australian Patterns of Tobacco Smoking in 1986,  D.J. Hill,  MJA, Vol 149, Jul 4, 1988  Pierce (1989). International Comparisons of Trends in Cigarette Smoking Prevalence. American Journal of Public Health, Vol. 79, No.2, Pg. 152-157. |
| Cancer Council Victoria (CCV) Survey | 1989 | n=4,829  Persons aged 16+ years living in Australia. | An interview at home | Not available | Unknown. | A random sample survey was conducted in 11 electoral subdivision, which were chosen at random with 13 strata that were based upon 5 states and the Australian Capital Territory and Northern Territory, as split in rural and urban categories. A part of an omnibus survey.  Data collector: a large market-research company – a name was not stated. | Australian Patterns of Tobacco Smoking in 1989,  D.J. Hill, V.M. White and N.J. Gray, MJA, Vol 154, Jun 17, 1991 |
| 00794 – The Risk Factor Prevalence Survey (RFPS) | 1989 | n=9,279  Aged 20-69, residents of Australian capital cities. | A self-completed questionnaire (distribute, collect) accompanied by a clinical test | 74.7%  (Note: this is a combined response rate across 1980, 1983 and 1989 RFPS) (Please see: AIHW 2003. Indicators of health risk factors: the AIHW view. AIHW Cat. No. PHE 47. Canberra: AIHW). | YES | All electors on Commonwealth Electoral Rolls as at 31 December, 1988. The catchment areas were in Sydney North, Sydney South, Melbourne, Brisbane, Adelaide, Perth, Hobart, Darwin and Canberra.  **Collection situation**  Letters of invitation were sent to participants approximately 2 weeks prior to the set appointment date, with a reminder telephone call given the day before. Participants who did not respond to the original letter were either re-invited by telephone or sent a second letter of invitation. If a response was still not received after the second invitation and the participant could not be telephoned, they were then visited by an experienced interviewer from the Australian Bureau of Statistics.  On the day of the appointment, participants completed a questionnaire and then underwent a physical examination, including a blood sample.  A joint National Heart Foundation of Australia and AIHW publication. | ADA  https://dataverse.ada.edu.au/dataset.xhtml?persistentId=doi:10.26193/UEKW8Q  https://legacy.ada.edu.au/social-science/00794  https://www.aihw.gov.au/reports/heart-stroke-vascular-diseases/rfps-survey-no-3-1989-cities-analysis/contents/summary |
| National Health Survey | 1989-90 | n=54,576 | Personal interviews | 96.1 | YES | Households were selected at random using a stratified multi-stage area sample which ensured a representation of persons within each State and Territory. | https://www.abs.gov.au/AUSSTATS/abs@.nsf/Lookup/4366.0Main+Features11989-90?OpenDocument  https://www.abs.gov.au/AUSSTATS/abs@.nsf/productsbyCatalogue/615974DC68259544CA256EB4007C3E92?OpenDocument |
| 00610 - National Campaign Against Drug Abuse Social Issues in Australia Survey (NCADASIS) | 1991 | n=2,850  Persons aged 14+ years. | A face-to-face interview and self-completed questionnaire | Not available | NO | Persons living in private dwellings and caravan parks, in Australian towns or cities of at least 5,000 people.  Sampling procedure: stratified area-cluster sample with age-sex quota  Conducted by: Wells Research | ADA  https://dataverse.ada.edu.au/dataset.xhtml?persistentId=doi:10.26193/K2EJPV  http://legacy.ada.edu.au/social-science/00610 |
| Cancer Council Victoria (CCV) Survey | 1992 | n=6,046  Persons aged 16+ years living in Australia. | A face-to-face interview at home | Not available | Unknown. | A random sample survey was conducted in 11 electoral subdivision, which were chosen at random with 13 strata that were based upon 5 states and the Australian Capital Territory and Northern Territory, as split in rural and urban categories. A part of an omnibus survey.  Data collector: a large market-research company – a name was not stated. | Australian Adult Smoking Prevalence in 1992, D.J. Hill and V.M. White, Australian Journal of Public Health, Vol 19, No. 3, 1995 |
| 00820 – Victorian Drug Household Survey (VDHS) | 1993 | n=600  Household persons aged 14+ in Victoria. | Personal interview and a self-completed questionnaire | 49% | YES | A survey undertaken in parallel with National Drug Strategy Household Surveys. In 1993 and 1995, additional interviews were conducted in Victoria using a Victoria-specific questionnaire which included questions of particular interest to Victorian Government organisations.  Estimates of age by sex for the Victorian population based on ABS projections in 1991 were used to weight the data.  Multi-stage stratified random sample.  Survey conductor: AGB McNair consultancy firm. | ADA  http://legacy.ada.edu.au/social-science/00820 |
| 00765 - National Campaign Against Drug Abuse Social Issues in Australia Survey (NCADASIS) | 1993 | n=3,500  A household member aged 14+. | A face-to-face interview and self-complete questionnaire | Not available | YES | Except for the ACT, 70% of each state sample was allocated to metropolitan areas and 30% to rural areas. | ADA  https://dataverse.ada.edu.au/dataset.xhtml?persistentId=doi:10.26193/55CBCR  http://legacy.ada.edu.au/social-science/00765 |
| Cancer Council Victoria (CCV) Survey | 1995 | n=5,699  Persons aged 17+ years living in Australia. | A face-to-face interview at home | 47% | Unknown. | A random sample survey was conducted in 11 electoral subdivision, which were chosen at random with 13 strata that were based upon 5 states and the Australian Capital Territory and Northern Territory, as split in rural and urban categories. A part of an omnibus survey.  Data collector: a large market-research company – a name was not stated. | Smoking Behaviours of Australian Adults in 1995: Trends and Concerns, D.J. Hill, V.M. White and M.M. Scollo, MJA, Vol. 168, 209-213. |
| 00979 – Victorian Drug Strategy Household Survey (VDSHS) | 1995 | n=1,200  (600 from NDSHS survey/ 600 from Victoria-specific only survey).  Household persons aged 14+ in Victoria | Personal interview with a confidential self-completion section | 55% a national survey/ 56% the Victorian survey | YES | A survey undertaken in parallel with National Drug Strategy Household Surveys. In 1993 and 1995, additional interviews were conducted in Victoria using a Victoria-specific questionnaire which included questions of particular interest to Victorian Government organisations.  Stratified random sample.  Survey conductor: AGB McNair consultancy firm | ADA  http://legacy.ada.edu.au/social-science/00979 |
| 00862 - National Drug Strategy Household Survey (NDSHS) | 1995 | n=3,850  Persons aged 14+ years. | A face-to-face interview and sealed section of a questionnaire (self-completed).  Data Collector: AGB McNair | 57% | YES  A two-stage weighting process was undertaken in order to calculate person specific weights. The first stage involved calculating the probability of the selection of the person within the household, to account for the higher possibility of selecting certain persons from smaller households. The second stage involved an age/sex expansion weight to ensure the age/sex estimated benchmarks for each state were achieved according to current population based on the 1991 ABS Census counts. | Geographical unit: Urban/Rural by State (smaller islands excluded).  One-time cross-sectional study - partial replication. Multi-stage stratified random sample. | ADA  https://dataverse.ada.edu.au/dataset.xhtml?persistentId=doi:10.4225/87/GF5LNY  http://legacy.ada.edu.au/social-science/00862 |
| 01016 - National Drug Strategy Household Survey (NDSHS) | 1998 | n=10,340  Persons aged 14+ years. | 1. Drop and collect:  Sample 1: Personal Interview and Self Completion Questionnaire Sample 2: Distribute and Collect Sample 3: Distribute and Collect  A personal interview and self-completion  methodology adopted in past surveys, also incorporated two additional samples using variants of a self-completion methodology. All three samples were collected over the same period, namely from the end of June to mid-September 1998. Data from a total of 10,030  respondents are included in the final results  Sample 1: Personal Interview and Self Completion Questionnaire; Sample 2: Distribute and Collect (sample 1 and Sample 2 could be collected at the same time from a same household vs Sample 3 that were collected from different households to Sample 1 and 2 and in metropolitan areas only and among 14-39 years only) Sample 3: Distribute and Collect.  Data Collector: Roy Morgan Research Centre | 56% | YES  Four different sets of weights were calculated: - Sample 1 alone - Sample 1 and Sample 2 combined - Sample 3 alone - All three samples combined The sample was designed to provide (within each geographic stratum) a close-to-random sample of households. The selection of S1 and S3 respondents was designed to be unbiased. (The selection of the S2 respondents followed a different procedure, with a deliberate bias towards younger persons and is discussed separately below.) However, the resulting samples required weighting to correct for imbalances arising in the design and execution of the sampling. Each respondent was assigned a weight designed to counteract this imbalance overall. Where estimates are derived from the sample they are based on the weighted samples unless stated otherwise. The disproportionate sampling by region meant that it was necessary to attach lower weights to respondents from relatively over-sampled regions and higher weights to respondents from relatively under-sampled regions. Households were selected with equal probability, meaning that the probability of selection of an individual was inversely proportional to the number of persons aged 14+ in the household, this probability being taken into account in the calculation of the individual's weight, so that respondents in households of different sizes were represented in their due proportions. As an initial check on the sample composition, within each stratum the selected Census collector Districts (CCDs) were compared with all CCDs on a number of measures, using the results from the 1996 census. This revealed no material bias in the selection of CCDs. For detailed information on the weights for each sample and final adjustments to weights, please, refer to Section 6 of the Technical Report (see Related Materials). | Geographical unit: Urban/Rural by State (smaller islands excluded).  A total of 10,699 households were called upon across Australia in order to complete a Sample One interview. Contact was actually made at 7,355 of these households. This represents a contact rate of 69% (up from 67% in 1995). The final response rate for Sample 1 was 55% (completed, useable questionnaires as a percentage of households contacted).  The final response rate for Sample 2 was 61% (completed, usable questionnaires as a percentage of Sample One households with more than one person aged 14 or over).  The final response rate for Sample 3 was 60% (completed, usable questionnaires as a percentage of households that accepted a questionnaire). Many Sample 3 questionnaires were deemed unusable after being returned as they were totally blank, insufficiently complete or sent in by respondents who were out of the specified age range (not aged between 14 and 39 years).    A multi-stage stratified sample design was used.  The first year that the Australian Institute of Health and Welfare (AIHW) has been collating and reporting on these surveys. | ADA  https://dataverse.ada.edu.au/dataset.xhtml?persistentId=doi:10.4225/87/XJE0KQ  http://legacy.ada.edu.au/social-science/01016 |
| 01050 - National Drug Strategy Household Survey (NDSHS) | 2001 | n=26,744  Persons aged 14+ years. | 1. Drop & Collect: ‘Face-to-face’ interview and self-completed questionnaire. For the 'Drop & collect' sample, a sealed section of the questionnaire allowed respondents to indicate their usage of each drug without the interviewer being aware of their answers.  2. Telephone interview (CATI): For the Computer Assisted Telephone Interview (CATI) sample these sections were asked directly by the interviewer.  A total of 22,649 complete and useable questionnaires were obtained using the self-completion drop-and-collect method.  A total of 2,055 interviews were completed using the face to face personal interview method.  A total of 2,040 interviews were completed using the CATI method.  Data Collector : Roy Morgan Research | Overall response rate: 50%  CATI: 49%  Drop &Collect: 51%  Personal ‘Face to Face’ interview: 39% | YES  A number of different calculations were made as part of the weighting process, in general, the published data has been published utilising the weights for all three samples combined. For detailed information on weighting for each of the three samples and the weighting for the 'combined sample', please, see sections 6.4 and 7.3 of the National Drug Strategy Household Survey 2001 Technical Report.  https://dataverse.ada.edu.au/file.xhtml?fileId=680&version=1.1 | Geographical unit: Urban/Rural by State.  Stratification process: A multi-stage stratified sample design was used. The sample was stratified by region in line with the Institute’s requirements and included over-sampling in some States and Territories. | ADA  https://dataverse.ada.edu.au/dataset.xhtml?persistentId=doi:10.4225/87/DZIPIJ  http://legacy.ada.edu.au/social-science/01050 |
| National Health Survey | 2001 | n=26,863 | Personal interview | 92% | YES | The NHS was conducted in a sample of 17,918 private dwellings across Australia. Both urban and rural areas in all states and territories were included, but sparsely settled areas of Australia were excluded. Non private dwellings such as hotels, motels, hostels, hospitals, nursing homes and short-stay caravan parks were not included in the survey. | https://www.abs.gov.au/AUSSTATS/abs@.nsf/allprimarymainfeatures/0E3F9F6786419026CA25711F0008D22F?opendocument |
| 01082 - National Drug Strategy Household Survey (NDSHS) | 2004 | n=29,445  Persons aged 12+ years. | 1. Drop & Collect: For the 'Drop & collect' sample, a sealed section of the questionnaire allowed respondents to indicate their usage of each drug without the interviewer being aware of their answers.  2. Telephone interview (CATI): For the Computer Assisted Telephone Interview (CATI) sample these sections were asked directly by the interviewer.  A booster sample of those aged 12-29 was conducted in Queensland using the Drop & Collect methodology.  Data Collector: Roy Morgan Research | Overall response rate: 46%  CATI: 38%  Drop & Collect: 48% | YES  For a detailed description of weighting please refer:  https://dataverse.ada.edu.au/file.xhtml?fileId=674&version=1.1 | Geographical unit: Urban/Rural by State.  Sampling procedure:  Drop and Collect - Multi-stage stratified random sample. CATI- Simple random sample.  A multi-stage stratified random sample design was used. The sample was stratified by region in line with the Institute’s requirements and included over-sampling in some States and Territories.  Overall, contact was made with 50,426 in-scope households selected for the Drop & Collect sample, of which, the total of 24,109 complete and useable questionnaires represented 48%. This is the response rate that is used, in this report, to compare the rates  between the two data collection methods. | ADA  https://dataverse.ada.edu.au/dataset.xhtml?persistentId=doi:10.4225/87/M3IFO8  http://legacy.ada.edu.au/social-science/01082 |
| National Health Survey | 2004/05 | n= 25,906 | Personal interview | 86.4%  household response rate | YES | Participants were from urban and rural areas across all States and Territories of Australia; those from very remote areas were excluded from the sample.  Survey was conducted using a stratified multistage area sample of private dwellings. | https://www.abs.gov.au/AUSSTATS/abs@.nsf/allprimarymainfeatures/B03BAF3CACBFE1A8CA2577E30018F6D4?opendocument  https://www.abs.gov.au/ausstats/abs@.nsf/7d12b0f6763c78caca257061001cc588/bb16f1c1ebed7695ca257633001d2e46!OpenDocument |
| 01139 - National Drug Strategy Household Survey (NDSHS) | 2007 | n=23,356  Persons aged 12+ years. | 1. CATI – Residents of Australian households with telephones.  2. Drop and Collect – Residents (self-completed?) of Australian households.  The 2007 Survey utilised self-completion (Drop & Collect) and Computer Assisted  Telephone Interviewing (CATI) methodologies as adopted in past surveys. The CATI interviewing was conducted by a second consultant, the Social Research Centre. The Drop  & Collect methodology was conducted by Roy Morgan Research from 19 June to 23 October 2007. Responses returned by reply paid envelopes were accepted until 28 October 2007. Data from a total of 23,356 respondents (19,818 from the Drop and Collect component and 3,538 from the CATI component) are included in the final results.  Data Collector:  1. Australian Institute of Health and Welfare (Drop & Collect). 2. Social Research Centre (CATI). | Overall rate: 49.3%  CATI – 39.3%  Drop & Collect-51.6%. | YES  For a detailed description of weighting please refer:  https://dataverse.ada.edu.au/file.xhtml?fileId=656&version=1.1 | Geographical unit: Urban/Rural by State.  Sampling procedure:  Drop and Collect - Multi-stage stratified random sample. CATI- Simple random sample.  A multi-stage stratified random sample design was used. The sample was stratified by region (15 strata in total - Capital City and rest of State, with the ACT operating as one stratum) in line with the Institute’s requirements, and included over-sampling in some States and Territories.  Overall, contact was made with 36,698 in-scope households selected for the Drop & Collect sample, of which, the total of 19,818 complete and useable questionnaires represented 54%.  Conducted between July and November of 2007. | ADA  https://dataverse.ada.edu.au/dataset.xhtml?persistentId=doi:10.4225/87/T7FITH  http://legacy.ada.edu.au/social-science/01139  https://www.aihw.gov.au/reports/illicit-use-of-drugs/2007-ndshs-first-results/contents/table-of-contents |
| National Health Survey | 2007-08 | n=20,788 | A face-to-face interview | 91% | YES | Private dwellings only. Urban and rural areas. All States and Territories; additional sample in Northern Territory. | https://www.abs.gov.au/ausstats/abs@.nsf/cat/4364.0  https://www.abs.gov.au/ausstats/abs@.nsf/7d12b0f6763c78caca257061001cc588/bb16f1c1ebed7695ca257633001d2e46!OpenDocument |
| 01237 – National Drug Strategy Household Survey (NDSHS) | 2010 | n=26,648  Persons aged 12+ years. | 1. Drop-and-Collect methodology only (self-completed?).  Data collector: Roy Morgan Research | 50.6% | YES  Weighting method: Imbalances due to sampling procedure, response rates, under-sampled states, and over sampled states and territories are adjusted for with the weighting variables for each stratum. Main weighting takes into account geographical stratification, household size, age and sex based on the latest published ABS estimated resident population data | Geography: Australia National – all states and territories.  Geographical unit: Census Collection District.  Residential households. The scope excluded non-private dwellings (hotels, motels, boarding houses, etc.) and institutional settings (hospitals, nursing homes, other clinical settings such as drug and alcohol rehabilitation centres, prisons, military establishments and university halls or residence). Homeless persons were also excluded as well as the territories of Jervis Bay, Christmas Island and Cocos Island. For the first time very remote locations were not deemed inaccessible in all cases, with field workers flown to these locations.  A multi-stage stratified random sample design was used. The sample was stratified by region (15 strata in total – Capital City and rest of State, with the ACT operating as one stratum). Contact was attempted with a total of 81,708 households Australia-wide. Of these 52,690 were contacted and in-scope. Questionnaires were successfully placed at 37,566 households (representing 46% of households at which contact was attempted, or 71% of in-scope households at which contact was made).  Overall, contact was made with 52,690 in-scope households, from which 26,648 complete and usable questionnaires were received, representing a response rate for the 2010 survey of 51%.  Conducted between April and September of 2010. | ADA  https://dataverse.ada.edu.au/dataset.xhtml?persistentId=doi:10.4225/87/VTUV4C  http://legacy.ada.edu.au/social-science/01237 |
| National Health Survey | 2011-12 | n= 20,426 | A face-to-face interview | 86%  household response rate | YES | Private dwellings only. Urban and rural areas. All States and Territories; additional sample in Northern Territory. | https://www.abs.gov.au/AUSSTATS/Abs@.Nsf/Latestproducts/9E22DA7218BCCADACA257B8D00229EA5?opendocument |
| 01274 – National Drug Strategy Household Survey (NDSHS) | 2013 | n=23,855  Persons aged 12+ years. | 1. Drop-and-Collect methodology only (self-completed?). This method was previously used in 2010, 2007, 2004, 2001 and 1998, but added an additional third pick-up attempt to improve response rates.  Data collector:  Roy Morgan Research | 49.1% | YES  Weighting method: The survey was designed to provide (within each geographic stratum) a close-to-random sample of households within the scope of the respective samples. The selection of respondents within households was designed to be unbiased. However, the resulting samples still required weighting, to correct for imbalances arising from execution of the sampling and differential response rates, and to bring the under-sampled and over-sampled strata back in line with the population. Where survey estimates are derived from the sample they should be based on the weighted results unless specified otherwise. The weight used in calculation of the statistics in the NDSHS codebook is Weight 7, the Absolute Person Weight. This weight (also known as the Single Respondent Weight) encompasses the components of within-household selection probability, non-response adjustment and stratum balancing. It should be noted that a number of different weights were required to be calculated as part of the weighting process. The details of the weighting process and the various weights created and included with this data are specified on p.28-33 of the NDSHS Technical Report (see Files tab). | Geography: all states and territories.  Geographical unit: Census Collection District.  Residential households. The scope excluded non-private dwellings (hotels, motels, boarding houses, etc.) and institutional settings (hospitals, nursing homes, other clinical settings such as drug and alcohol rehabilitation centres, prisons, military establishments and university halls or residence). Homeless persons were also excluded as well as the territories of Jervis Bay, Christmas Island and Cocos Island.  Of an initial sample of 75,992 households, 3006 were identified as out of scope, and 24,407 could not be contacted. Of the 48,579 households contacted and in scope, 13,945 were refused, while a further 1,662 eligible but unable to take part due to other reasons (such as language difficulties).  Conducted between July and November of 2013. | ADA  http://legacy.ada.edu.au/social-science/01274  https://dataverse.ada.edu.au/dataset.xhtml?persistentId=doi:10.4225/87/USGEQS |
| National Health Survey | 2014-15 | n=19,259 | A face-to-face interview | 82%  household response rate | YES | Usual residents of private dwellings in urban and rural areas in all States and Territories and across urban, rural and remote areas of Australia (other than very remote areas).  The 2014-15 NHS was conducted using a stratified multistage area sample of private dwellings. | https://www.abs.gov.au/ausstats/abs@.nsf/Lookup/by%20Subject/4363.0~2014-15~Main%20Features~Structure%20of%20the%20National%20Health%20Survey~10 |
| 01389 - National Drug Strategy Household Survey (NDSHS) | 2016 | n=23,772  Persons aged 12+ living in Australia. | 1. Drop and collect survey (paper form).  2. Telephone interview.  3.Web-based self-completion.  Data collector:  Roy Morgan Research | 51.1% | YES  Weighting method: The sample was designed to provide a random sample of households within each geographic stratum. Respondents within each stratum were assigned weights to overcome imbalances arising in the design and execution of the sampling. The main weighting took into account geographical stratification, household size, age and sex. The population estimates used for the weighting were based on the latest available age/sex profile using the latest published ABS Estimated Resident Population data (Cat. no. 3101.0 - – Australian Demographic Statistics, June 2016). The 3101.0 series provided gave the necessary level of age breakdown by State/Territory territory but not by stratum. The stratum level population estimates were projected from the ABS Population by Age and Sex, Regions of Australia series (Cat no. 3235.0 – Population by Age and Sex, Regions of Australia, 2015). All estimates should be based on the weighted sample. Main weight is Weight7. Use Weight8 for household weighting. | Geography: all states and territories.  Residential households. The scope excluded non-private dwellings (hotels, motels, boarding houses, etc.) and institutional settings (hospitals, nursing homes, other clinical settings such as drug and alcohol rehabilitation centres, prisons, military establishments and university halls or residence). Homeless persons were also excluded as well as the territories of Jervis Bay, Christmas lsland and Cocos lsland  Sample description: Size of original (target) sample: 70,935. Non-contacts and out of scope: 24,448. Refusals and other non-response 14,749. Incompletes: 7,966.  Conducted between June and November 2016. | ADA  https://dataverse.ada.edu.au/dataset.xhtml?persistentId=doi:10.4225/87/JUDY2Y |
| National Health Survey | 2017-18 | n=21,315 | Personal interview | 76% household response rate | YES | Conducted in all states and territories, urban, rural and remote areas (other than very remote areas). | https://www.abs.gov.au/ausstats/abs@.nsf/Lookup/by%20Subject/4363.0~2017-18~Main%20Features~Structure%20of%20the%20National%20Health%20Survey~10 |
