# Supplementary material for: Birth-cohort estimates of smoking initiation and prevalence in 20th century Australia: Synthesis of data from 33 surveys and 385,810 participants
Source: PLoS One. 2021 May 21;16(5):e0250824. doi: 10.1371/journal.pone.0250824 (PMC8139520; doi:10.1371/journal.pone.0250824)
Supplement: S2 Table — NHS Australian/National Health Survey NDSHS: National Drug Strategy Household Survey RFPS: Risk Factor Prevalence Study/Survey. (DOCX) [file pone.0250824.s008.docx]

| S2 Table. Mean age of smoking initiation among ever-smokers by sex and survey series for 10-year birth-cohort in Australia (excluding those surveyed at 30 years and younger). NHS Australian/National Health Survey NDSHS: National Drug Strategy Household Survey RFPS: Risk Factor Prevalence Study/Survey | | | | | | | | | | | | | | |
| --- | --- | --- | --- | --- | --- | --- | --- | --- | --- | --- | --- | --- | --- | --- |
|  | **MALES** | | | | | | | **FEMALES** | | | | | | |
|  | N | Mean  age  (year) | N | Mean  age  (year) | N | Mean  age  (year) | N | | Mean  age  (year) | N | Mean  age  (year) | N | Mean  age  (year) |  |
| **Birth-cohort** | **NHS** | | **NDSHS** | | **RFPS** | | **NHS** | | | **NDSHS** | | **RFPS** | |  |
| 1910-19 | 596 | 19.0 | 249 | 19.8 | 204 | 18.3 | 508 | | 28.1 | 102 | 23.4 | 122 | 24.0 |  |
| 1920-29 | 1,692 | 17.6 | 1,695 | 18.9 | 1,716 | 18.0 | 1,377 | | 22.0 | 876 | 22.4 | 919 | 22.9 |  |
| 1930-39 | 3,199 | 17.4 | 3,504 | 18.5 | 1,499 | 17.9 | 2,239 | | 21.0 | 2,115 | 22.2 | 902 | 21.3 |  |
| 1940-49 | 5,240 | 17.1 | 6,214 | 18.1 | 1,758 | 17.7 | 4,071 | | 19.4 | 4,966 | 20.2 | 1,235 | 19.3 |  |
| 1950-59 | 6,371 | 17.0 | 6,673 | 17.9 | 742 | 17.5 | 5,508 | | 18.0 | 6,455 | 19.0 | 574 | 18.7 |  |
| 1960-69 | 4,000 | 17.2 | 6,095 | 17.9 | - | - | 3,989 | | 17.4 | 7,857 | 18.0 | - | - |  |
| 1970-79 | 3,031 | 17.5 | 3,315 | 18.3 | - | - | 3,085 | | 17.2 | 4,445 | 18.0 | - | - |  |
| 1980-89 | 666 | 17.4 | 601 | 18.7 | - | - | 643 | | 16.9 | 697 | 17.8 | - | - |  |
