# Supplementary material for: Birth-cohort estimates of smoking initiation and prevalence in 20th century Australia: Synthesis of data from 33 surveys and 385,810 participants
Source: PLoS One. 2021 May 21;16(5):e0250824. doi: 10.1371/journal.pone.0250824 (PMC8139520; doi:10.1371/journal.pone.0250824)
Supplement: S3 Table — NHS Australian/National Health Survey NDSHS: National Drug Strategy Household Survey RFPS: Risk Factor Prevalence Study/Survey. (DOCX) [file pone.0250824.s009.docx]

S3 Table. Smoking initiation by age, sex and 10-year birth-cohort calculated from empirical distributions in Australia (excluding those surveyed at 30 years and younger). NHS Australian/National Health Survey NDSHS: National Drug Strategy Household Survey RFPS: Risk Factor Prevalence Study/Survey

|  |  | **Aged <=15**  **(%)** | | **Aged <=20**  **(%)** | | **Aged <=25**  **(%)** | | **Aged <=30**  **(%)** | | **Aged <=35**  **(%)** | | **Aged <=40**  **(%)** | |
| --- | --- | --- | --- | --- | --- | --- | --- | --- | --- | --- | --- | --- | --- |
|  |  | M | F | M | F | M | F | M | F | M | F | M | F |
| NDSHS | 1910-19 | 15.1 | 6.8 | 64.1 | 50.5 | 90.4 | 71.8 | 97.6 | 89.3 | 98.4 | 94.2 | 99.2 | 95.1 |
|  | 1920-29 | 16.0 | 6.0 | 82.1 | 52.7 | 94.5 | 76.2 | 96.9 | 86.1 | 97.9 | 90.3 | *98.4 | 94.5 |
|  | 1930-39 | 21.8 | 7.3 | 82.0 | 59.3 | 94.4 | 81.2 | 97.4 | 89.8 | 98.1 | 93.5 | 98.9 | 96.7 |
|  | 1940-49 | 23.2 | 11.4 | 85.0 | 71.5 | 95.3 | 88.3 | 97.9 | 94.4 | 98.8 | 96.9 | 99.2 | 98.1 |
|  | 1950-59 | 24.3 | 16.9 | 85.3 | 79.8 | 95.5 | 92.2 | 98.2 | 96.4 | 98.8 | 97.4 | 99.2 | 98.6 |
|  | 1960-69 | 27.0 | 24.5 | 84.0 | 84.8 | 94.3 | 94.7 | 97.4 | 97.5 | 98.5 | 98.7 | 99.5 | 99.5 |
|  | 1970-79 | 22.7 | 23.5 | 78.7 | 81.8 | 94.1 | 95.0 | 98.0 | 98.4 | 99.6 | 99.5 | 99.9 | 100.0 |
|  | 1980-89 | 13.1 | 22.8 | 78.0 | 84.8 | 94.0 | 95.8 | 99.0 | 99.3 | 100.0 | 100.0 | - | - |
|  |  | M | F | M | F | M | F | M | F | M | F | M | F |
| RFPS | 1910-19 | 21.8 | 5.2 | 75.9 | 48.4 | 96.6 | 66.0 | 98.5 | 86.9 | 99.2 | 92.8 | 100.0 | 96.7 |
|  | 1920-29 | 23.2 | 5.9 | 81.9 | 56.4 | 95.7 | 79.7 | 98.5 | 89.1 | 99.1 | 92.8 | 99.7 | 97 |
|  | 1930-39 | 26.1 | 7.2 | 82.5 | 61.2 | 95.9 | 83.2 | 98.8 | 92.2 | 99.3 | 95.3 | 99.8 | 98.3 |
|  | 1940-49 | 23.4 | 10.1 | 86.6 | 76.6 | 97.0 | 92.0 | 98.8 | 96.8 | 99.9 | 98.6 | 100.0 | 99.8 |
|  | 1950-59 | 23.6 | 9.5 | 87.8 | 79.1 | 96.9 | 95.9 | 99.3 | 99.4 | 100.0 | 100.0 | - | - |
|  |  | M | F | M | F | M | F | M | F | M | F | M | F |
| NHS | 1910-19 | 22.9 | 8.4 | 72.3 | 51.7 | 92.0 | 73.0 | 97.6 | 89.1 | 99.2 | 94.8 | 100.0 | 100.0 |
|  | 1920-29 | 23.5 | 9.2 | 83.2 | 58.9 | 96.3 | 81.3 | 98.9 | 91.2 | 99.6 | 95.6 | 100.0 | 100.0 |
|  | 1930-39 | 27.6 | 10.0 | 83.0 | 62.5 | 95.7 | 84.2 | 98.6 | 93.2 | 99.3 | 97.0 | 100.0 | 100.0 |
|  | 1940-49 | 28.1 | 13.9 | 86.5 | 74.0 | 96.8 | 90.6 | 99.0 | 96.3 | 99.6 | 98.7 | 100.0 | 100.0 |
|  | 1950-59 | 29.6 | 19.4 | 87.0 | 82.4 | 96.6 | 94.6 | 99.0 | 98.2 | 99.6 | 99.2 | 100.0 | 100.0 |
|  | 1960-69 | 30.7 | 27.7 | 85.0 | 86.0 | 95.3 | 95.5 | 98.3 | 98.1 | 99.3 | 99.1 | 100.0 | 100.0 |
|  | 1970-79 | 26.2 | 27.3 | 81.4 | 83.8 | 95.2 | 95.9 | 98.5 | 98.7 | 99.8 | 99.6 | 100.0 | 100.0 |
|  | 1980-89 | 21.4 | 28.8 | 82.7 | 87.5 | 96.1 | 96.9 | 99.5 | 99.6 | 100.0 | 100.00 | - | - |
|  | | | | | | | | | | | | | |
