# Supplementary material for: Birth-cohort estimates of smoking initiation and prevalence in 20th century Australia: Synthesis of data from 33 surveys and 385,810 participants
Source: PLoS One. 2021 May 21;16(5):e0250824. doi: 10.1371/journal.pone.0250824 (PMC8139520; doi:10.1371/journal.pone.0250824)
Supplement: S4 Table — NHS Australian/National Health Survey NDSHS: National Drug Strategy Household Survey RFPS: Risk Factor Prevalence Study/Survey. (DOCX) [file pone.0250824.s010.docx]

| S4 Table. Mean age of smoking initiation among ever-smokers by sex and 5-year birth-cohort (excluding those surveyed at 30 years and younger). NHS Australian/National Health Survey NDSHS: National Drug Strategy Household Survey RFPS: Risk Factor Prevalence Study/Survey | | | | | | | | | | | | | |
| --- | --- | --- | --- | --- | --- | --- | --- | --- | --- | --- | --- | --- | --- |
|  | **NDSHS (n=55,859)** | | | | | **RFPS (n=9,671** | | | | **NHS (n=33,147)** | | | |
| **Birth cohort** | **N** | | **Mean** | |  | **N** | | **Mean** | | **N** | | **Mean** | |
|  | M | F | M | F |  | M | F | M | F | M | F | M | F |
| <1919 | 251 | 103 | 19.8 | 23.3 |  | 266 | 153 | 18.2 | 23.6 | - | - | - | - |
| 1920-24 | 581 | 308 | 19.2 | 24.2 |  | 807 | 436 | 18.3 | 22.8 | 122 | 122 | 18.2 | 21.6 |
| 1925-29 | 1116 | 569 | 18.8 | 23.3 |  | 923 | 485 | 17.8 | 22.0 | 533 | 401 | 17.5 | 21.1 |
| 1930-34 | 1384 | 837 | 18.6 | 22.7 |  | 745 | 409 | 17.9 | 21.7 | 809 | 579 | 17.1 | 21.5 |
| 1935-39 | 2122 | 1282 | 18.5 | 21.9 |  | 733 | 488 | 17.8 | 20.9 | 1097 | 797 | 17.2 | 20.6 |
| 1940-44 | 2787 | 2103 | 18.3 | 20.8 |  | 821 | 583 | 17.9 | 19.8 | 1441 | 1060 | 16.6 | 19.6 |
| 1945-49 | 3429 | 2863 | 17.9 | 19.8 |  | 937 | 661 | 17.5 | 18.9 | 1841 | 1519 | 16.9 | 19.1 |
| 1950-54 | 3395 | 2938 | 18.0 | 19.5 |  | 481 | 371 | 17.9 | 19.0 | 1917 | 1552 | 16.9 | 18.8 |
| 1955-59 | 3289 | 3531 | 17.7 | 18.6 |  | 206 | 166 | 16.8 | 18.0 | 2038 | 1905 | 16.8 | 17.8 |
| 1960-64 | 3317 | 4098 | 17.8 | 18.1 |  | - | - | - | - | 2159 | 2032 | 17.1 | 17.4 |
| 1965-69 | 2759 | 3740 | 18.0 | 17.8 |  | - | - | - | - | 1841 | 1957 | 17.3 | 17.4 |
| 1970-74 | 2062 | 2899 | 18.3 | 18.0 |  | - | - | - | - | 1909 | 1929 | 17.5 | 17.2 |
| 1975-79 | 1253 | 1546 | 18.3 | 18.1 |  | - | - | - | - | 1122 | 1156 | 17.6 | 17.3 |
| 1980-84 | 546 | 632 | 18.7 | 17.9 |  | - | - | - | - | 556 | 554 | 17.4 | 16.9 |
| 1985-89 | 55 | 65 | 19.0 | 17.3 |  | - | - | - | - | 110 | 89 | 17.2 | 16.7 |
