# Supplementary material for: Birth-cohort estimates of smoking initiation and prevalence in 20th century Australia: Synthesis of data from 33 surveys and 385,810 participants
Source: PLoS One. 2021 May 21;16(5):e0250824. doi: 10.1371/journal.pone.0250824 (PMC8139520; doi:10.1371/journal.pone.0250824)
Supplement: S5 Table — NDSHS: National Drug Strategy Household Survey RFPS: Risk Factor Prevalence Study/Survey *Australian/National Health Survey was excluded due to small numbers in some birth cohorts. (DOCX) [file pone.0250824.s011.docx]

|  | S5 Table. Smoking initiation by sex, age and 5-year birth-cohort calculated from empirical distributions (excluding those surveyed at 30 years and younger). NDSHS: National Drug Strategy Household Survey RFPS: Risk Factor Prevalence Study/Survey *Australian/National Health Survey was excluded due to small numbers in some birth cohorts. | | | | | | | | | | | | |
| --- | --- | --- | --- | --- | --- | --- | --- | --- | --- | --- | --- | --- | --- |
|  |  | **Aged <=15** | | **Aged <=20** | | **Aged <=25** | | **Aged <=30** | | **Aged <=35** | | **Aged <=40** | |
|  |  | **(%)** | | **(%)** | | **(%)** | | **(%)** | | **(%)** | | **(%)** | |
|  |  | **M** | **F** | **M** | **F** | **M** | **F** | **M** | **F** | **M** | **F** | **M** | **F** |
| **NDSHS** | **1920-24** | 13 | 6 | 80 | 47 | 94 | 74 | 97 | 83 | 98 | 89 | 98 | 94 |
|  | **1925-29** | 18 | 6 | 84 | 56 | 95 | 78 | 97 | 88 | 98 | 91 | 98 | 95 |
|  | **1930-34** | 20 | 7 | 82 | 57 | 94 | 80 | 97 | 89 | 98 | 93 | 98 | 96 |
|  | **1935-39** | 23 | 8 | 82 | 61 | 94 | 82 | 98 | 90 | 98 | 94 | 99 | 97 |
|  | **1940-44** | 21 | 10 | 84 | 68 | 95 | 86 | 98 | 93 | 99 | 97 | 99 | 98 |
|  | **1945-49** | 25 | 12 | 86 | 74 | 96 | 90 | 98 | 95 | 99 | 97 | 99 | 98 |
|  | **1950-54** | 22 | 14 | 85 | 76 | 95 | 91 | 98 | 96 | 99 | 97 | 99 | 99 |
|  | **1955-59** | 27 | 19 | 86 | 83 | 96 | 93 | 98 | 97 | 99 | 98 | 99 | 99 |
|  | **1960-64** | 27 | 23 | 86 | 85 | 94 | 95 | 97 | 97 | 98 | 98 | 99 | 99 |
|  | **1965-69** | 27 | 27 | 82 | 85 | 94 | 95 | 98 | 98 | 99 | 99 | 100 | 100 |
|  | **1970-74** | 24 | 25 | 78 | 82 | 93 | 95 | 98 | 98 | 100 | 100 | 100 | 100 |
|  | **1975-79** | 20 | 21 | 80 | 82 | 96 | 95 | 99 | 99 | 100 | 100 | 100 | 100 |
|  | **1980-84** | 13 | 21 | 79 | 85 | 94 | 96 | 99 | 99 | 100 | 100 | 100 | - |
|  | **1985-89** | 11 | 39 | 71 | 88 | 93 | 94 | 100 | 100 | - | - | - | - |
|  |  | **M** | **F** | **M** | **F** | **M** | **F** | **M** | **F** | **M** | **F** | **M** | **F** |
| **RFPS** | **1920-24** | 19 | 5 | 81 | 52 | 96 | 79 | 99 | 88 | 90 | 92 | 94 | 97 |
|  | **1925-29** | 27 | 6 | 83 | 61 | 95 | 80 | 98 | 90 | 99 | 93 | 100 | 97 |
|  | **1930-34** | 28 | 6 | 81 | 56 | 96 | 82 | 99 | 91 | 99 | 95 | 100 | 99 |
|  | **1935-39** | 24 | 8 | 84 | 65 | 96 | 84 | 99 | 93 | 100 | 96 | 100 | 98 |
|  | **1940-44** | 23 | 10 | 85 | 73 | 96 | 90 | 98 | 95 | 100 | 98 | 100 | 100 |
|  | **1945-49** | 24 | 10 | 88 | 80 | 98 | 94 | 99 | 99 | 100 | 100 | 100 | 100 |
|  | **1950-54** | 21 | 7 | 86 | 76 | 96 | 95 | 99 | 99 | 100 | 100 | 100 | - |
|  | **1955-59** | 29 | 15 | 92 | 86 | 99 | 98 | 100 | 100 | - | - | - | - |
